# FUS toxicity is rescued by the modulation of lncRNA *hsrω* expression in *Drosophila melanogaster*

**DOI:** 10.1038/s41598-017-15944-y

**Published:** 2017-11-15

**Authors:** Luca Lo Piccolo, Salinee Jantrapirom, Yoshitaka Nagai, Masamitsu Yamaguchi

**Affiliations:** 1 Department of Applied Biology, The Center for Advanced Insect Research, Kyoto Institute of Technology, Matsugasaki, Sakyo-ku, Kyoto, 606-8585 Japan; 2 Department of Neurotherapeutics, Osaka University Graduate School of Medicine, 2-2 Yamadaoka, Suita, Osaka, 565-0871 Japan

## Abstract

FUS is an aggregation-prone hnRNP involved in transcriptional and post-transcriptional regulation that aberrantly forms immunoreactive inclusion bodies in a range of neurological diseases classified as FUS-proteinopathies. Although FUS has been extensively examined, the underlying molecular mechanisms of these diseases have not yet been elucidated in detail. We previously reported that RNAi of the lncRNA *hsrω* altered the expression and sub-cellular localization of *Drosophila* FUS in the central nervous system of the fly. In order to obtain a clearer understanding of the role of *hsrω* in FUS toxicity, we herein drove the expression of human FUS in *Drosophila* eyes with and without a *hsrω* RNAi background. We found that hFUS was largely soluble and also able to form aggregates. As such, hFUS was toxic, inducing an aberrant eye morphology with the loss of pigmentation. The co-expression of *hsrω* double-stranded RNA reduced *hFUS* transcript levels and induced the formation of cytoplasmic non-toxic hFUS-LAMP1-insoluble inclusions. The combination of these events caused the titration of hFUS molar excess and a removal of hFUS aggregates to rescue toxicity. These results revealed the presence of a lncRNA-dependent pathway involved in the management of aggregation-prone hnRNPs, suggesting that properly formed FUS inclusions are not toxic to cells.

## Introduction

FUS-proteinopathies are a group of genetically and clinically heterogeneous diseases that manifest in different manners depending on the region affected, such as motor neuron diseases (ALS-FUS) or various forms of dementia, including frontotemporal lobar degeneration with FUS pathology (FTLD-FUS), atypical FTLD with ubiquitin pathology (aFTLD-U), and other distinct forms of FTLD such as neuronal intermediate filament inclusion disease (NIFID) and basophilic inclusion body disease (BIBD)^1^. A hallmark of these conditions is immunoreactive FUS inclusion bodies^2–5^. Several missense mutations in the *FUS* gene have been associated with the abnormal cytoplasmic localization of pathological FUS inclusions that are positive for p62 and ubiquitin in amyotrophic lateral sclerosis (ALS); however, FUS mutations have not been detected in most sporadic or familial cases of FTLD-FUS^6^.

A large number of animal models have been employed to elucidate the underlying molecular mechanisms of FUS-proteinopathies, and *Drosophila melanogaster* has been utilized to discern some important aspects of their pathogenesis^7–11^ due to the conservation of FUS protein functions during evolution. All phenotypes caused by the removal of the *Drosophila* FUS orthologue Cabeza (dFUS), such as decreased adult viability, locomotor disabilities, and a short life span may be rescued by the heterologous expression of human FUS (hFUS)^10,12–15^. Knockdown models and the cell-type specific inactivation of dFUS have both contributed to our understanding of the important role of FUS in neuronal development and also highlight how the loss of its nuclear function may induce the defects typically observed in some FUS-proteinopathies^11,12,15,16^. Moreover, heterologous expression in the fly revealed that hFUS has the ability to translocate to mitochondria through the mediation of Hsp60 and induces apoptosis as a consequence of mitochondrial damage^17^. This recent finding showed that in the absence of mutations, higher FUS levels also represent a critical event in FUS-proteinopathies. Consistent with this finding, sequencing of the 3′-untranslated region (3′-UTR) of *FUS* mRNA in ALS patients, with no mutations in the currently known ALS-associated genes, identified four mutations linked to a marked increase in the accumulation of FUS^18^, indicating that not only the nuclear–cytoplasmic imbalance of the mutant FUS, but also alterations in the physiological levels of the wild-type (wt) contribute to the pathogenesis of ALS.

Research over the past decade has revealed that FUS is a RNA-binding protein of the FUS, EWSR1, and TAF15 (FET) family, is 526 amino acids long, and is characterized by an N-terminal domain enriched in glutamine, glycine, serine, and tyrosine residues (QGSY), an RNA-recognition motif (RRM), multiple RGG-repeat regions involved in RNA binding, a C2/C2 zinc finger motif, and a highly conserved C-terminal region^19,20^. FUS is intrinsically prone to aggregate^21^ and plays multiple nuclear and cytoplasmic steps in RNA processing^6^, thereby regulating the spatiotemporal fate of mRNA, i.e. subcellular localization, translation, or degradation^22,23^. FUS has been associated with general and more specialized factors that influence the initiation of transcription, splicing regulation, micro-RNA processing, and, more recently, the formation of circular RNAs^24^. FUS may be co-transcriptionally deposited onto nascent mRNAs and drive RNA decay, which maintains this interaction during mRNA transport to the cytoplasm^22^. FUS has the ability to control its own levels through a potent auto-regulatory mechanism, and, thus, the overexpression of FUS negatively affects its own expression post-transcriptionally^25,26^. FUS preferentially binds to long transcripts, and previous studies highlighted its ability to specifically control a large number of long non-coding RNAs (lncRNAs) with important functions at different levels in the central nervous system (CNS)^27^. On the other hand, lncRNAs are dysregulated upon the depletion or unavailability of functional FUS in diverse models and diseases^28^, which raises questions about how FUS-proteinopathies affect lncRNA-based mechanisms.

Despite the large number of new findings, further studies are required in order to obtain a better understanding of the pathogenesis of FUS-proteinopathies. Although the progression of these diseases may be attributed to the propagation of FUS misfolding and aggregation^29^, previous studies also reported that FUS has an inherent ability to form diverse and distinct insoluble partitions under physiological conditions^30,31^. Hence, the mechanisms by which cells manage aggregation-prone proteins and how FUS inclusions correlate with diseases are not completely understood. Moreover, although an increasing number of studies have clearly revealed the important role of lncRNAs in many neurological diseases, it has not yet been established whether they also play a role in the pathogenesis of FUS-proteinopathies.

We recently reported that the RNAi of the *Drosophila* lncRNA *hsrω* negatively controlled the abundance of the *dFUS* transcript and altered the dFUS nuclear-cytoplasmic balance^32^. This was the first study to show the lncRNA-based control of *FUS* transcription and, until now, it represents the first evidence for the sub-cellular localization of FUS being dependent on lncRNAs.

With the main aim to understand the role of *hsrω* in FUS-proteinopathies, we herein drove the expression of hFUS in *Drosophila* eyes with and without a *hsrω* RNAi background. When expressed in the posterior region of the imaginal disc, hFUS was distributed in two fractions: mainly soluble and also able to form relative soluble aggregates. As such, hFUS was toxic, inducing an aberrant eye morphology with the loss of pigmentation in the corresponding adult flies. The co-expression of *hsrω* double-stranded RNA (dsRNA) rescued hFUS toxicity through a double mechanism. The depleted *hsrω* transcript induced a decrease in *hFUS* mRNA levels and altered its solubility to form hFUS-LAMP1-insoluble inclusions. The combination of these events ultimately led to the titration of a hFUS molar excess and rescued toxicity. We found that neither proteasome, autophagy, nor lysosomes were involved in the degradation of hFUS-LAMP1 inclusions, which makes it more difficult to clarify the role of the lysosome-associated membrane protein LAMP1 and also suggests that these inclusions are specifically targeted to be a deposit as safe cytoplasmic particles.

These novel results revealed an evolutionarily conserved mechanism to control *FUS* transcripts based on lncRNA and suggest a new role for LAMP1 in the formation of cytoplasmic hFUS deposits. The present results also contribute to our understanding of the pathomechanism of FUS-proteinopathies and indicate that a lncRNA-dependent mechanism may manage aggregation-prone hnRNPs such as FUS in non-toxic inclusions.

## Results

### hFUS expressed by the eye disc-specific GMR-Gal4 driver is largely soluble and toxic

The expression of hFUS driven by the eye disc-specific GMR-Gal4 system causes alterations in compound eye morphology^9,16,17,33^. In the present study, we used the hFUS transgenic flies described previously^34^. In order to set our experimental conditions, we compared the effects of hFUS expression on the eye external surface of 1- and 6-day-old adult flies carrying *GMR*;+ ;*GFP* and *GMR*;*hFUS*;+ developed at 25 and 28 °C, respectively (Figure S1). Male and female hFUS-expressing flies at 25 °C showed an area of degeneration with fused ommatidia only 6 days after eclosion, while an aberrant eye structure was clearly detected in 1-day-old adult flies developed at 28 °C. The most severe neurodegeneration was consistently found in 6-day-old flies by driving the expression of hFUS at 28 °C. Under these conditions, male and female flies showed fused ommatidia and a wide area in which pigmentation was lost. However, we observed bristle overproduction in males and its underproduction in females. We then drove the expression of hFUS at a higher temperature, and, thus, unless otherwise stated, flies used in subsequent experiments were developed at 28 °C.

**Figure 1:**
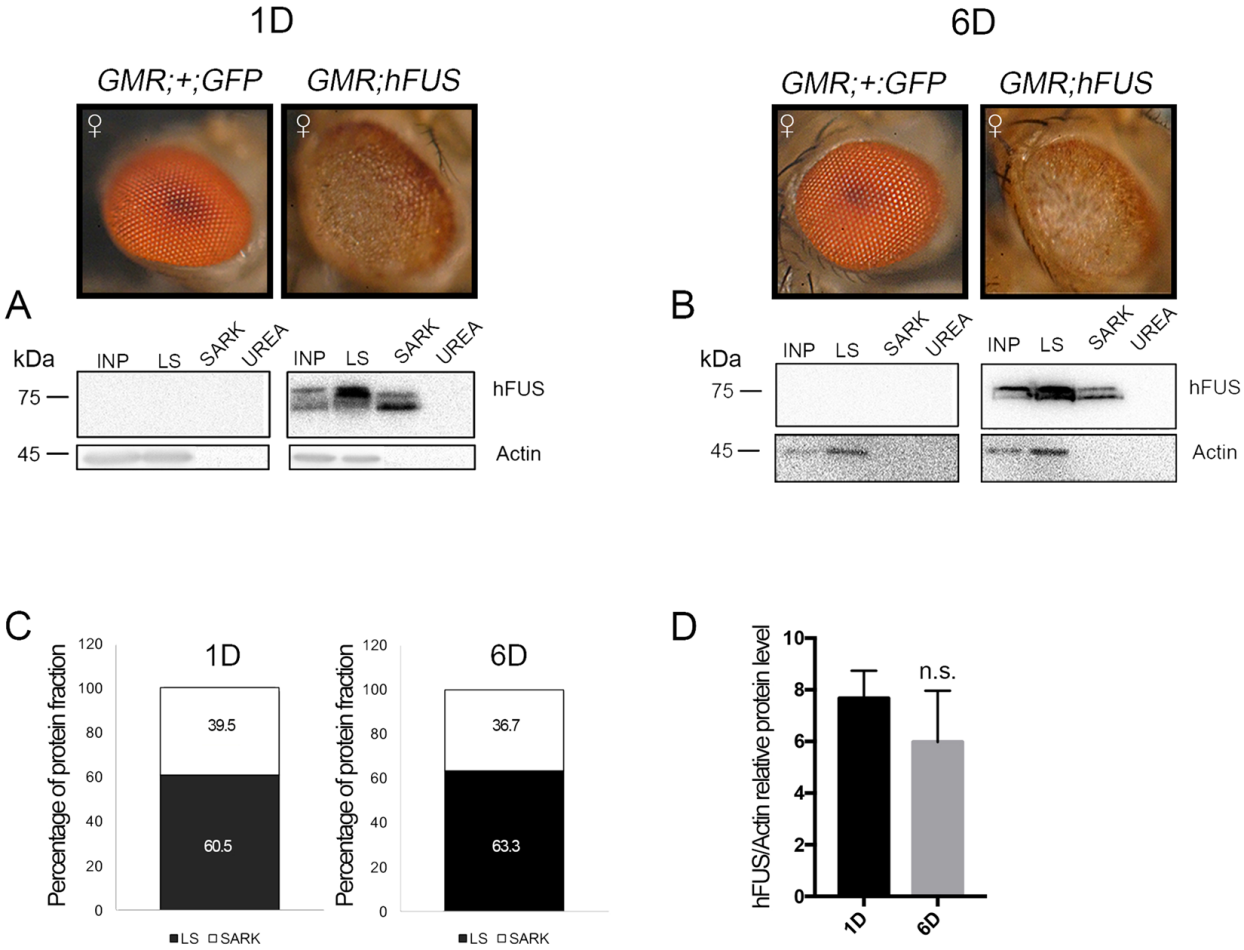
hFUS expressed by the eye disc-specific GMR-Gal4 driver is mainly soluble. (**A**,**B**) Light microscopy of compound eyes of 1 and 6 days-old flies carrying *GMR-GAL4*;+ *:*UAS*-GFP* and *GMR-GAL4*;UAS *-hFUS*;+ developed at 28 °C, respectively. The eye pictures are adapted from the Figure S1. Solubility test on hFUS extracted from *Drosophila* adult compound eyes by the GMR-Gal4 driver. Western blot analyses were performed with an anti-hFUS antibody using proteins fractioned with Low Salt buffer (LS), 2% N-Lauroylsarcosine-containing buffer (SARK), and 8 M Urea-containing buffer (UREA) from the adult heads of 1 (1D)- and 6 (6D)-day-old flies after eclosion. An Input (INP) of fractionation was analyzed on 10% SDS-PAGE to normalize the relative distribution of hFUS in the different fractions. “Percentage of protein fraction” indicates the protein levels in individual fractions normalized to input. Gel plots of the hFUS electrophoretic pattern profile were obtained with ImageJ32 software and used to measure the hFUS abundance in each fraction. (**C**) The bar charts show the relative percentage of hFUS fractions. (**D**) hFUS protein abundance was estimated relative to actin abundance. Protein extraction was performed in duplicate and two Western blots were independently performed with each sample. Twenty micrograms of the crude extract was loaded onto 10% SDS-PAGE. Gel plots were obtained with ImageJ32 and GraphPad Prism 7.0 software was used to statistically analyze data (n = 4). (n.s.) not significant.

It currently remains unclear whether the toxicity of hFUS is associated with its ability to form aggregates. Therefore, in order to examine this putative connection, we performed a biochemical fractionation of proteins extracted from the adult heads of *GMR*;*hFUS*;+ -expressing flies (Fig. 1). The solubility of hFUS has been studied in detail in human culture cells^35–39^; however, only two previous studies performed a solubility test on hFUS expressed in flies^9,33^. In these studies, fractionation was performed using RIPA buffer to collect soluble proteins and UREA- or SDS-containing buffer to recovery insoluble fractions. In order to distinguish between aggregates and inclusions, we herein investigated the solubility of hFUS in three different solutions by adapting a method of a previous study on the solubility of TDP43^40^, another aggregation-prone hnRNP involved in proteinopathies. Accordingly, the soluble fraction was extracted in law salt buffer (LS), relative soluble aggregates were separated using N-Lauroylsarcosine-containing buffer (SARK), and the insoluble fraction was recovered in urea-containing buffer (UREA). We found that hFUS was largely present in the LS fraction, with no significant difference being observed in percentages between samples obtained from 1- and 6-day-old flies (60.5 and 63.3%, respectively) (Fig. 1C). Although hFUS was mainly soluble, it was also fractioned in SARK (39.5 and 36.7%, respectively) (Fig. 1C and Figure S4, white arrows), indicating that when expressed in flies, hFUS also forms relative soluble aggregates. We did not detect hFUS in the UREA fraction (Fig. 1A and B). We also measured the abundance of hFUS in the input (INP) of fractioning assays and found no significant difference in proteins extracted from 1- and 6-day-old *Drosophila* adult heads, respectively (Fig. 1D). Based on these results, we performed further experiments using 6-day-old flies only.

### hFUS regulates the expression and solubility of endogenous dFUS

**Figure 2:**
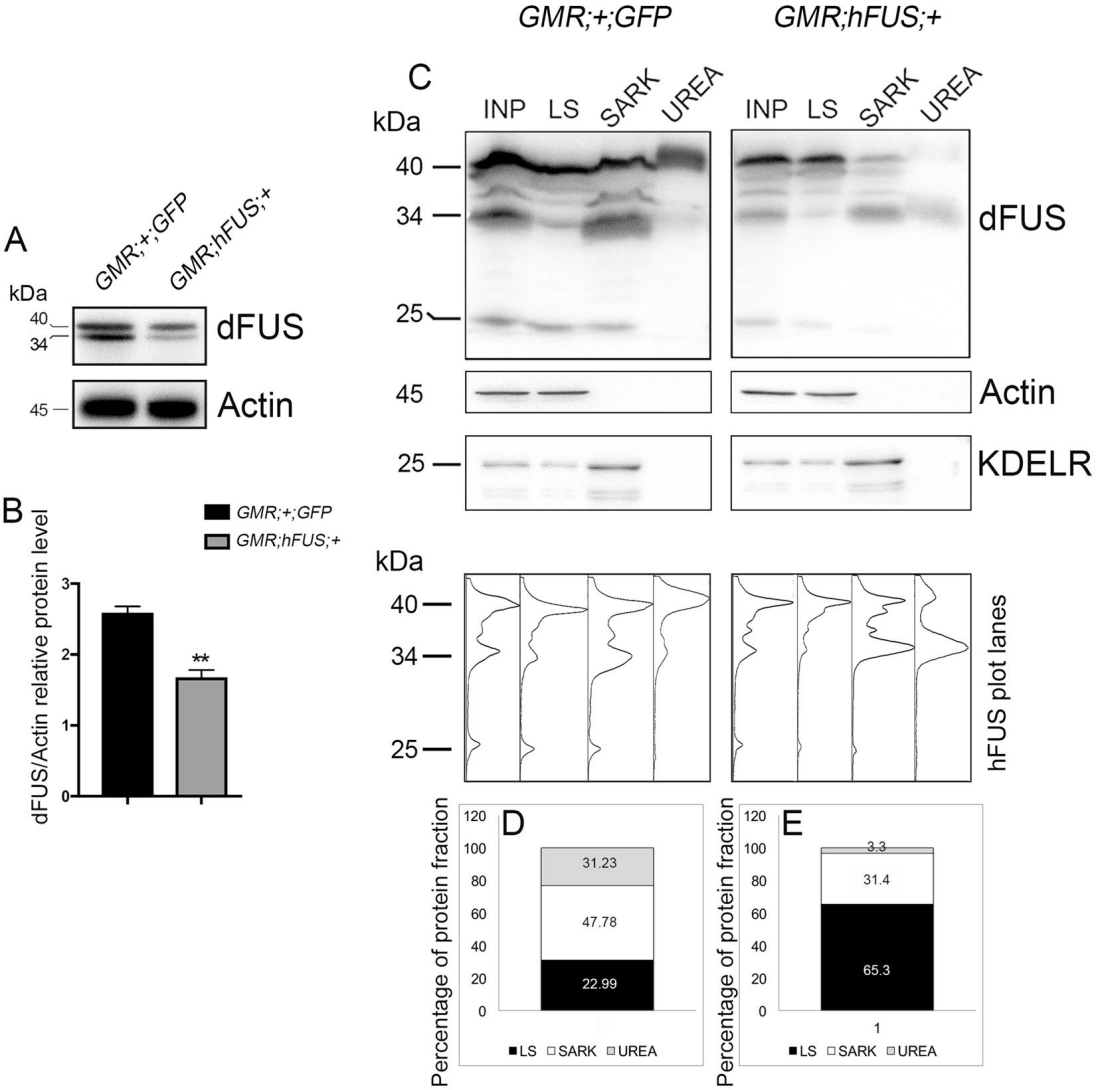
Abundance and solubility of endogenous dFUS are altered by hFUS expression. **(A**,**B**) Western blot analyses with an anti-dFUS antibody were performed using proteins extracted with lysis buffer from the adult heads of 6-day-old flies developed at 28 °C after eclosion. Two independent protein extractions, each loaded in duplicate for a total of four western blots, were statistically analyzed with GraphPad Prism 7.0. dFUS protein levels were normalized with respect to those of actin (**p-value < 0.005). (**C**) Western blot analyses were performed with an anti-dFUS antibody using proteins extracted with Low Salt buffer (LS), 2% N-Lauroylsarcosine-containing buffer (SARK), and 8 M Urea-containing buffer (UREA) from the adult heads of 6-day-old flies developed at 28 °C. An Input (INP) was also loaded onto 15% SDS-PAGE to normalize the relative distribution of dFUS in the different fractions. “Percentage of protein fraction” indicates the protein levels in individual fractions normalized to input. The fractionation study of Actin and KDELR was carried out to understand whether all the proteins were properly fractionated and recovered. (**D**,**E**) Gel plots of the hFUS electrophoretic pattern profile were obtained with ImageJ32 software and used to measure the dFUS abundance in each fraction. The bar charts show the relative percentage of dFUS fractions in control and hFUS-expressing flies.

Previous findings on the effects of hFUS expression on endogenous dFUS are contradictory^9,11,33^. In order to clarify whether hFUS expression exerts feedback on dFUS expression, we examined dFUS protein abundance and solubility (Fig. 2A–E). We performed these experiments using proteins extracted with lysis buffer from the adult heads of 6-day-old flies carrying *GMR*;+ ;*GFP* and *GMR*;*hFUS*;+ . We detected two major bands of dFUS: the highest 40-kDa and smaller 34-kDa (Fig. 2A). According to the predicted molecular weight of dFUS, we analyzed the relative abundance of the highest molecular weight carrying protein (Fig. 2B) and found a significant reduction (<1.76 fold, p-value < 0.05). Notably, dFUS34 was less abundant than the control. These results confirmed that hFUS expression driven by eye disc-specific GMR-Gal4 triggers negative feedback on endogenous dFUS expression. However, a reduction of dFUS amount may not contribute to the hFUS toxicity because previous studies have shown that dFUS knockdown can induce a mild degeneration of adult eye compounds^41^ while the phenotype herein observed was more similar to the aberrations caused by dFUS overexpression^9^.

We also investigated the effects of hFUS on dFUS solubility (Fig. 2C–E). The results obtained showed that in control dFUS was mainly fractionated in SARK (47.78%) and also distributed as 22.99% and 31.23% in LS and UREA fractions, respectively (Fig. 2D). The expression of hFUS markedly changed dFUS solubility, making endogenous dFUS mainly soluble (65.3% in LS) (Fig. 2E). Actin and the endoplasmic reticulum protein retention receptor (KDELR) were used as controls to understand whether all the proteins were properly and reproducibly fractionated and recovered. (Fig. 2E).

By scanning the bands detected in the Western blot assay, we observed that hFUS expression also affected the dFUS40:dFUS34 ratio (Fig. 2C, lowest panels). In the SARK fraction of control flies the dFUS40:dFUS34 ratio was an average of 1.29:1, while it was an average of 1:2.04 in the SARK fraction of flies carrying *GMR*;*hFUS*;+ . An altered dFUS40:dFUS34 ratio was also revealed in the UREA fraction because it shifted from 39.65:1 in the control to 1:7.69 in flies carrying *GMR*;*hFUS*;+ . Although the biological meaning of this alteration currently remains unknown, previous studies on TDP43 demonstrated that the smaller TDP25 is insoluble and toxic because it sequesters RNAs, which induces neurodegeneration^42^. Therefore, the augmentation of the smaller insoluble dFUS34 may play a role or contribute to the neurodegeneration induced by hFUS expression in flies. Cumulatively, these results extend the overview of hFUSopathies in fly models.

### *hsrω* RNAi rescues the hFUS-dependent rough eye phenotype in a dosage-dependent manner

**Figure 3:**
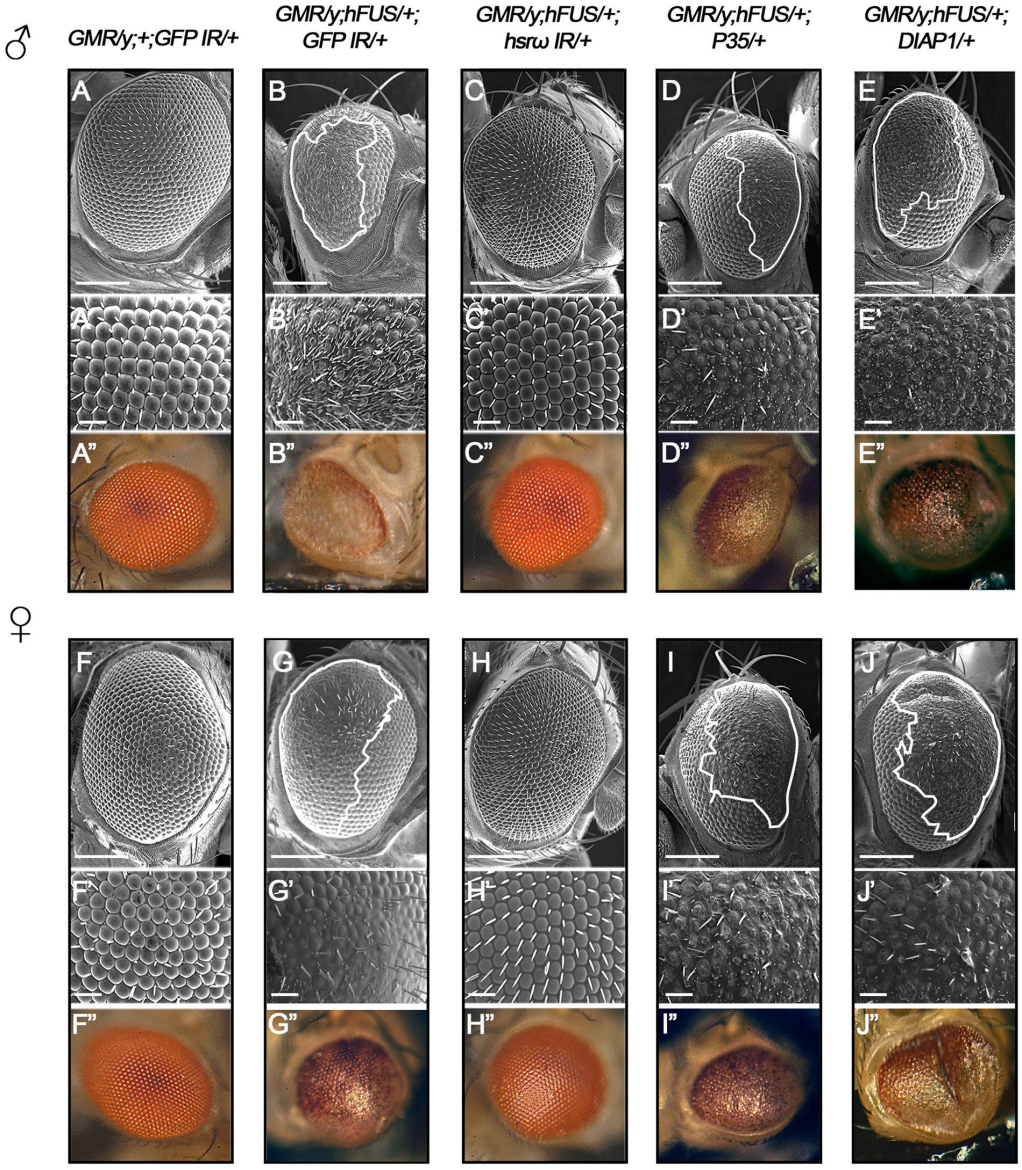
h*srω* RNAi rescues hFUS toxicity. Scanning electron micrographs and light microscopy of adult compound eyes with the following genotypes: (**A-A”**) *GMR-GAL4/*+;+ ;UAS*-GFP IR/*+, (**B-B”**) *GMR-GAL4/*+;UAS*-hFUS/*+;UAS*-GFP IR/*+, (**C-C”**) *GMR-GAL4/*+;UAS*-hFUS/*+;UAS*-hsrω IR/*+, (**D-D”**) *GMR-GAL4/*+;UAS*-hFUS/*+;UAS*-P35/*+, (**E-E”**) *GMR-GAL4/*+;UAS*-hFUS/*+;UAS*-DIAP1/*+. All flies were developed at 28 °C. The eye phenotypes of approximately 120 male and 130 female flies aged 6 days old from each line were examined under a light microscope, and the most representative ones were analyzed using SEM. Upper panels (A–E) and (F–J) (scale bar 50 µm). Middle panels (A’-E’) and (F’-J’) show a higher magnification (Scale bar: 14.2 µm). The lowest panels show the eye observed with a light microscope. No significant variation was observed in the eye phenotype among the individuals belonging to each line. Anterior is to the left and dorsal to the top. White lines define the area of degeneration.

Since we previously reported that the lncRNA *hsrω* physically and genetically interacted with dFUS^32^, we herein examined whether this type of interaction is evolutionary conserved by analyzing the effects of *hsrω* depletion on the aberrant eye morphology induced by hFUS. Flies carrying *GMR/*+;*hFUS/*+;*hsrω IR/*+appeared to have a normal eye morphology, indicating that *hsrω* RNAi rescued the toxicity induced by the expression of hFUS (Fig. 3, compare B and C).

Recent studies reported that the augmentation of hFUS induced apoptosis in flies due to its interaction with mitochondrial Hsp60^16,17^. Since *hsrω* is a regulator of apoptosis^43^, we initially hypothesized that this rescue was dependent on the inhibition of apoptosis triggered by *hsrω* RNAi. When we quantified cleaved caspase-3 (CC3)-positive cells in the posterior region of eye imaginal discs, a strong reduction in the immunoreaction was revealed (Figure S2). In flies carrying *GMR/*+;*hFUS/*+;*hsrω IR/*+, the number of CC3-positive cells was 66.67% less than that in flies carrying *GMR/*+;*hFUS/GFP IR*;+ , indicating that the depletion of *hsrω* reduces the number of apoptotic cells in hFUS-expressing flies. However, the expression of the P35 and DIAP1 anti-apoptotic factors driven by GMR-Gal4 in hFUS-expressing flies did not rescue eye morphology defects (Fig. 3, compare B, D and E). Flies carrying *GMR/*+;*hFUS/*+;*P35/*+and *GMR/*+;*hFUS/*+;*DIAP1/*+both showed fused ommatidia, the loss of bristles, and a wide area of degeneration, similar to those observed in *GMR/*+;*hFUS/GFP IR*;+ flies (Fig. 3D,E and Figure S3). The expression of P35 contributed to the partial amelioration of the eye surface, with only an 8.75% less degenerated area than *GMR/*+;*hFUS/GFP IR*;+ (Figure S3). These results indicate that the regulation of apoptosis by *hsrω* RNAi is not the major mechanism rescuing hFUS toxicity.

**Figure 4:**
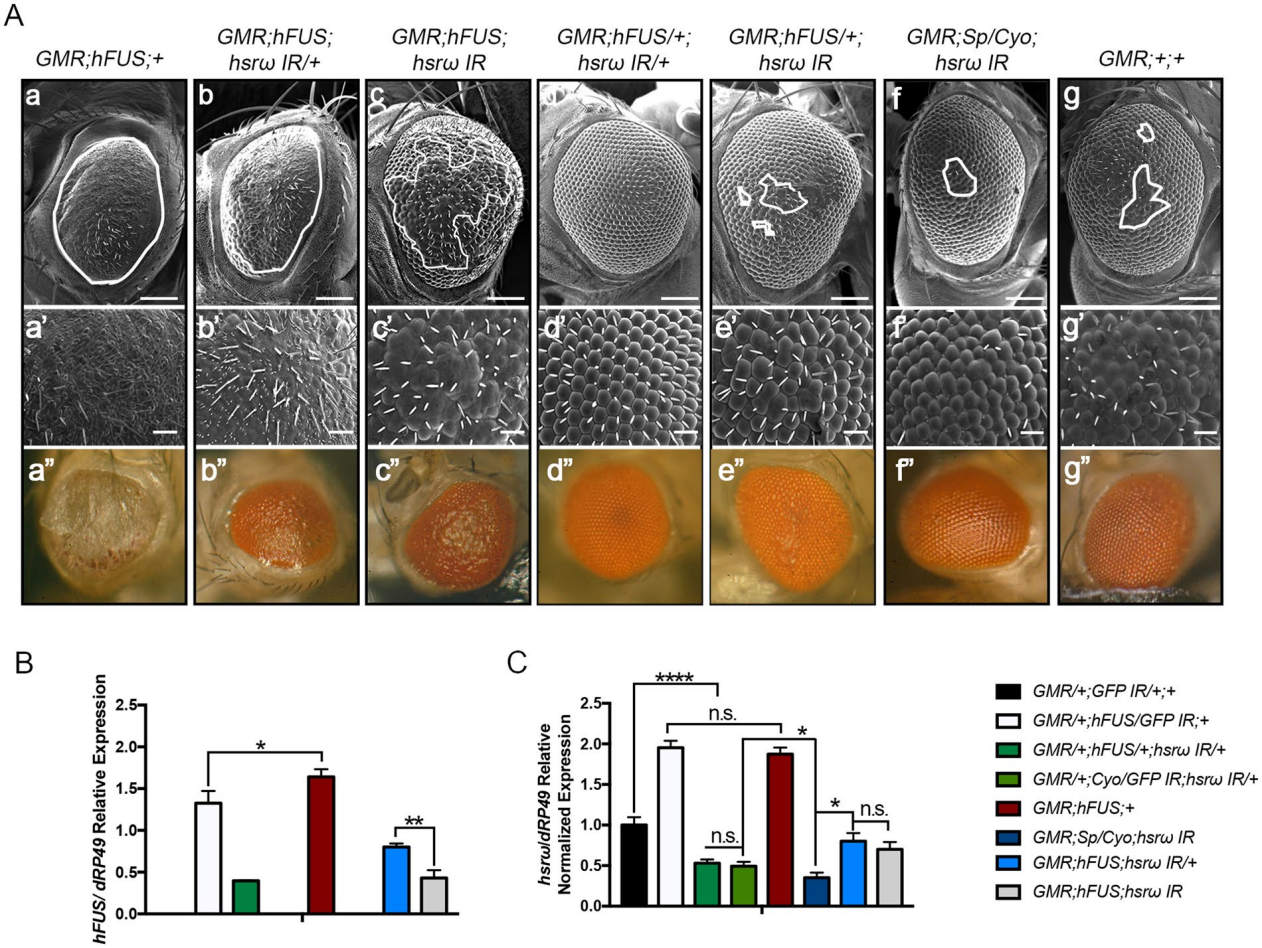
Fine gene expression regulation underlies the rescue of hFUS toxicity. **(A**) Scanning electron micrographs and light microscopy of adult compound eyes with the following genotypes: (**a-a”)** *GMR-GAL4*;UAS*-hFUS*, (**b-b”)** *GMR-GAL4*;UAS*-hFUS*;UAS*- hsrω IR/*+, (**c-c”**) *GMR-GAL4*;UAS*-hFUS*;UAS*-hsrω IR*, (**d-d”)** *GMR-GAL4*;UAS*-hFUS/*+;UAS*-hsrω IR/*+, (**e-e”)** *GMR-GAL4*;UAS*-hFUS/*+; UAS*-hsrω IR*, (**f-f”)** *GMR-GAL4*;+ ;UAS*-hsrω IR*, (**g-g”)** *GMR-GAL4*;+ ;+ . All flies were developed at 28 °C. The eye phenotypes of 100 male and 100 female flies aged 6 days old from each line were examined under a light microscope, and the most representative ones were analyzed using SEM. Upper panels (a–g) (scale bar 50 µm). Middle panels (a’-g’) show a higher magnification (Scale bar: 14.2 µm). The lowest panels show the compound eye observed with a light microscope. Anterior is to the left and dorsal to the top. White lines indicate the area of degeneration. Bigger and smaller white scale bars are 50 and 14.2 µm, respectively. (**B**,**C**) Quantification of transcript abundance. RNAs were extracted from the eye imaginal discs of the third instar larvae of different fly lines at three independent times and further analyzed by real-time RT-PCR in triplicate. The levels of the *hFUS* and *hsrω* transcripts were normalized with respect to *dRP49* abundance. Each transcript is the average of nine separate reactions for each fly line. A statistical analysis was performed using GraphPad Prism 7.0 software. *p-value < 0.05; ****p-value < 0.0001. n.s. = not significant.

We then examined the effects of the dosage of *hsrω* RNAi on hFUS toxicity by analyzing the eye phenotypes of homo- and heterozygous flies co-expressing hFUS mRNA and *hsrω* dsRNA, respectively (Fig. 4). The area of eye degeneration in homozygous flies carrying *GMR*;*hFUS*;+ (Fig. 4Aa) was gradually reduced by increasing the number of *hsrω* RNAi from single to double copies, as observed in flies carrying *GMR*;*hFUS*;*hsrω IR/*+ and *GMR*;*hFUS*;*hsrω IR* (Fig. 4Ab and Ac, respectively). Complete rescue was observed when a single copy of *hsrω* dsRNA was co-expressed with a single copy of *hFUS*, as shown in Fig. 4Ad and previously in Figure 3C. These results indicated that fine regulation underlies the interaction between *hsrω* and hFUS and also revealed that *hsrω* depletion affects hFUS toxicity in a hFUS dosage-dependent manner. In addition, we previously have reported that a knockdown of *hsrω* is critical to induce a reduction of *dFUS* transcript amount^32^ and we herein found that flies co-expressing hFUS and *hsrω* dsRNA show a normal eye phenotype to support the idea that hFUS toxicity in the fly may not due to the negative feedback triggered by hFUS on the endogenous dFUS.

A mild rough eye phenotype was also observed in flies carrying *GMR*;*hFUS/*+;*hsrω IR* (Fig. 4Ae), in which the area of degeneration and eye morphology were similar to those observed in *GMR*;+ ;*hsrω IR* flies (Fig. 4, compare Ae and Af). Consistent with previous findings^44^, *hsrω* RNAi exhibited the ability to rescue the aberrant eye structure caused by GMR-Gal4 (Fig. 4, compare Af and Ag); therefore, the phenotype observed in Fig. 4F and F may simply reflect rescue from the GMR-dependent rough eye background and does not appear to be related to the hFUS-*hsrω* interaction.

We also performed an extensive gene expression analysis with real-time RT-PCR to measure *hFUS* and *hsrω* transcripts (Fig. 4B and C) using RNA extracted from the eye imaginal discs of third instar larvae. Accordingly, *hFUS* mRNA levels were significantly higher in homozygous flies *GMR*;*hFUS*;+ than in heterozygous *GMR/*+;*hFUS/GFP IR*;+ flies, with an increment of 27% (Fig. 4B, compare white and red bars). Moreover, the depletion of *hsrω* was gradually stronger from one to two copies of *hsrω* RNAi, with 44.3% and 63.5% reductions in the *hsrω* transcript in heterozygous *GMR/*;*Cyo/GFP IR*;*hsrω IR/*+ and homozygous *GMR*;*Sp/Cyo*;*hsrω IR* flies, respectively, from that in control flies (Fig. 4C, compare light green and dark blue bars). A 66.89% reduction in the *hFUS* transcript was noted in samples of flies carrying *GMR/*+;*hFUS/*+;*hsrω IR/*+ (Fig. 4B, dark green bar), indicating that *hsrω* RNAi strongly reduces the *hFUS* transcript. This result is interesting also on the light of our previous finding to show likely the *dFUS* transcription is negatively affected by *hsrω* RNAi^32^.

In turn, the expression of hFUS in *Drosophila* eyes allowed a 50.28% increase in the *hsrω* transcript (Fig. 4C, white bar), with no significant differences being observed in flies carrying one or two copies of the *hFUS* transgene (Fig. 4C, white and red bars, respectively). We previously reported that the *hsrω* transcript increased when dFUS was down-regulated^32^, and, as described above, GMR-driven hFUS expression negatively affected dFUS expression (Fig. 2A and B). Hence, we hypothesized that the augmented transcription of *hsrω* observed in flies carrying *GMR/*+;*hFUS/GFP IR*;+ may depend on the down-regulation of dFUS induced by hFUS transgene expression. However, the increased *hsrω* abundance may not be involved in hFUS toxicity as discussed below (Figure S10).

Collectively, these results provide an insight into fine gene regulation between FUS and the lncRNA *hsrω* and support hFUS toxicity being completely or partly abolished as a result of the titration of the *hFUS* transcript induced by *hsrω* RNAi.

### *hsrω* RNAi shifts the solubility of hFUS and induces the formation of hFUS-LAMP1 inclusions

**Figure 5:**
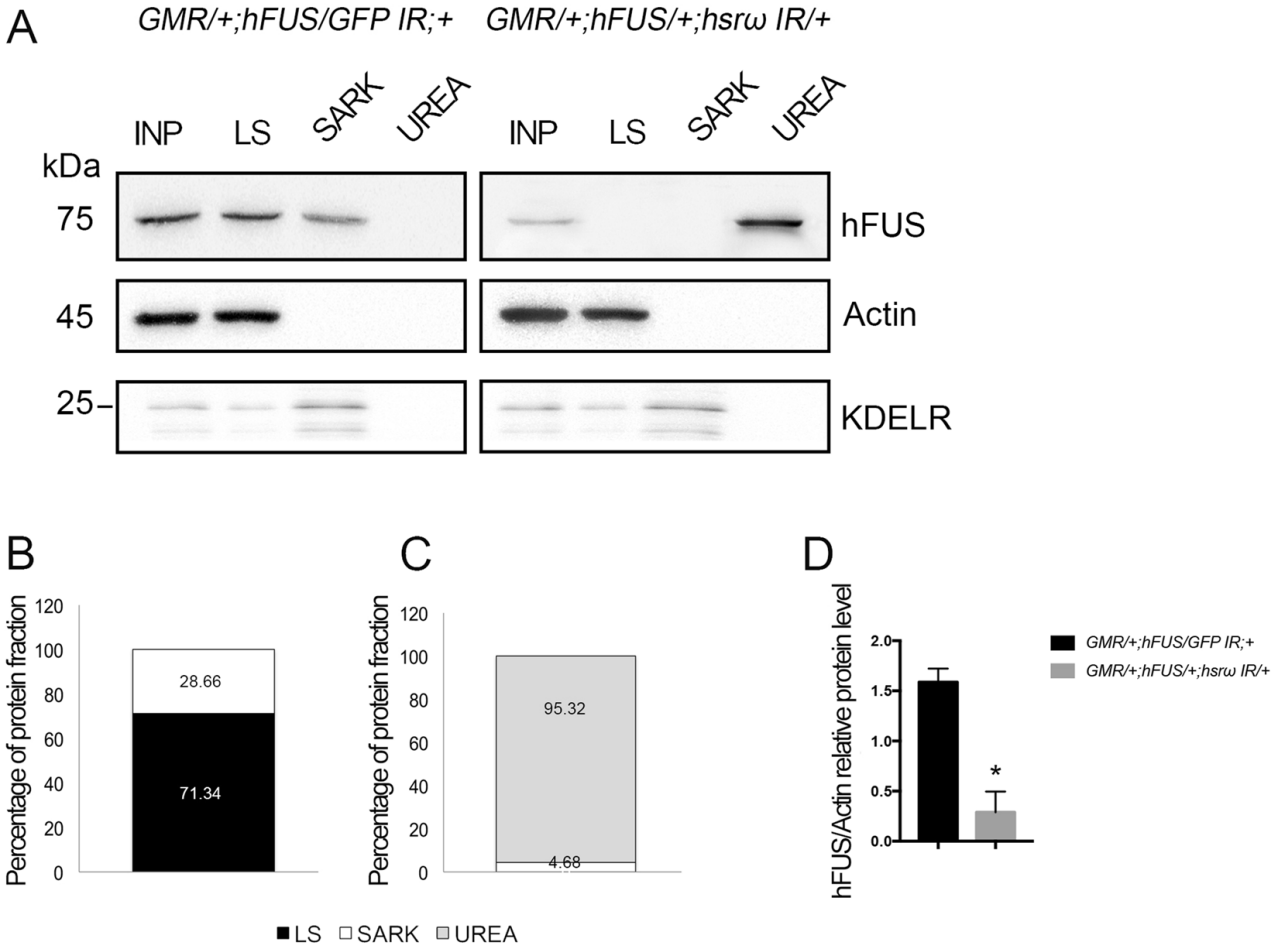
h*srω* RNAi shifts hFUS solubility. (**A–C**) A Western blot analysis with anti-hFUS was performed using proteins extracted from the adult heads of 6-day-old flies after eclosion with the following genotypes: *GMR-GAL4/*+;UAS*-hFUS/*+;UAS*-GFP IR/*+ and *GMR-GAL4/*+;UAS*-hFUS/*+;UAS*-hsrω IR/*+. Flies were developed at 28 °C. A solubility test was performed with a sequential combination of low salt (LS), 2% N-Lauroylsarcosine-containing (SARK), and 8 M urea-containing (UREA) buffers. An Input (INP) was also loaded onto 10% SDS-PAGE in order to normalize the relative distribution of hFUS in the different fractions. “Percentage of protein fraction” indicates the protein levels in individual fractions normalized to input. The fractionation study of Actin and KDELR was carried out to understand whether all the proteins were properly fractionated and recovered. Gel plots of the hFUS electrophoretic pattern profile were obtained with ImageJ32 software and used to measure hFUS abundance in each fraction. Bar charts show the relative percentages of hFUS fractions. (**D**) hFUS protein levels were measured relative to actin abundance. Fractionation was performed in duplicate using two independent protein extractions. Western blots were conducted in duplicate for each independent fractionation. Twenty micrograms of each fraction was loaded onto each 10% polyacrylamide gel. A statistical analysis was performed using GraphPad Prism 7.0 software. (n = 4). *p-value < 0.05.

In order to fully understand the mechanisms responsible for hFUS toxicity rescue, we studied potential modifications in the intracellular pattern of hFUS caused by *hsrω* RNAi. We performed a biochemical fractionation of *Drosophila* adult head proteins extracted from flies carrying *GMR/*+;*hFUS/GFP IR*;+ and *GMR/*+;*hFUS/*+;*hsrω IR/*+. We found that the depletion of lncRNA *hsrω* markedly altered hFUS solubility because hFUS in flies carrying *GMR/*+;*hFUS/*+;*hsrω IR/*+ was largely fractioned in UREA-containing buffer (95.32%), while 71.34% of hFUS in *GMR/*+;*hFUS/GFP IR*;+ flies was abundant in the LS fraction and completely absent in the UREA fraction (Fig. 5A–C). A statistical analysis on the relative amount of hFUS (input of fractioning, INP) showed a 67.36% reduction in *GMR/*+;*hFUS/*+;*hsrω IR/*+ flies from that in *GMR/*+;*hFUS/GFP IR*;+ flies (Fig. 5D).

The immunostaining of hFUS appeared to be consistent with its biochemical characterization. While hFUS in flies carrying *GMR/*+;*hFUS/GFP IR*;+ showed a wide distribution in the posterior region of eye imaginal discs with a dominant cytoplasmic localization of large and randomly sized hFUS-immunoreactive spots (Figure S4f and f’), a smaller area of the hFUS immunoreaction with a distinct punctate cytoplasmic pattern was revealed in flies carrying *GMR/*+;*hFUS/*+;*hsrω IR/*+ (Figure S4i and i’). Thus, in flies co-expressing hFUS mRNA and *hsrω* dsRNA, weak staining appeared to be due to the smaller amount of hFUS and the diverse hFUS-immunoreactive arrangement may agree with its insolubility.

As discussed above, since it currently remains unclear whether hFUS toxicity depends on its ability to form insoluble particles, these results represent an important step towards clarifying this issue. Based on the results in flies co-expressing hFUS mRNA and *hsrω* dsRNA showing that the rescue of toxicity coincides with both the establishment of insoluble hFUS particles and the loss of hFUS relative soluble aggregates, we concluded that hFUS inclusions are not toxic; their formation may represent a protective response that is controlled by the lncRNA *hsrω* in flies. On the other hands, these findings support the hFUS relative soluble aggregates may play the major role in hFUS toxicity.

A small amount of hFUS may simply reflect a reduction in *hFUS* transgene expression caused by down-regulated *hsrω*, as described above. hFUS solubility shifts have two potential biological meanings: it may be accomplished by the activation of a protein degradation pathway and insoluble hFUS formation may be a requisite for the activation of defense mechanisms. In order to clarify these possibilities, we initially examined the expression and solubility of proteasome components. Real-time RT-PCR was conducted with RNA extracted from the eye imaginal discs of third instar larvae carrying *GMR/*+;*hFUS/GFP IR*;+ and *GMR/*+;*hFUS/*+;*hsrω IR/*+ in order to compare the gene expression profiles of *Hsp70b and Hsp70c* genes with those of control larvae carrying *GMR/*+;*GFP IR/*+;+ and *GMR/*+;*Cyo/GFP IR*; *hsrω IR/*+ (Figure S5A). Increased levels of these genes were revealed in samples extracted from flies co-expressing hFUS mRNA and *hsrω* dsRNA. However, this up-regulation may depend more on the depletion of lncRNA *hsrω* than on a specific mechanism induced by the response to hFUS toxicity. A similar degree of up-regulation was detected when RNA samples from *GMR/*+;*Cyo/GFP IR*; *hsrω IR/*+ *-*expressing flies were analyzed (Figure S5A).

A Western blot analysis with anti-Hsp70 and anti-ubiquitin antibodies was performed using protein fractions obtained from *GMR/*+;*hFUS/GFP IR*;+ and *GMR/*+;*hFUS/*+;*hsrω IR/*+ flies and compared with *GMR/*+;*GFP IR/*+;+ control flies (Figure S5B). No significant differences were detected in the distribution of Hsp70 or ubiquitin among the different samples. Hsp70 was highly abundant in LS, while ubiquitin was enriched in SARK (Figure S5B–D). Globally, the ubiquitin electrophoretic pattern was the same in both samples, with the exception of an increase in the abundance of high molecular complexes (HMCs) and mono-ubiquitin (Ub) in the SARK fraction of *GMR/*+;*hFUS/*+;*hsrω IR/*+ flies. A statistical analysis confirmed the up-regulation of Hsp70 (Figure S5C) and monoUb-SARK in flies co-expressing hFUS mRNA and *hsrω* dsRNA (Figure S5D). Importantly, neither Hsp70 nor ubiquitin was detected in the UREA fraction, indicating that insoluble hFUS was ubiquitin-negative in this fly line and also that Hsp70 and ubiquitin were not hFUS-interacting proteins. Moreover, double immunostaining of the eye imaginal discs of third instar larvae confirmed the absence of an interaction between hFUS and Hsp70 (Figure S6).

These results supported the increased expression of proteasome components in flies co-expressing hFUS mRNA and *hsrω* dsRNA not being related to a solubility shift in hFUS. On the other hand, these results suggested that even if the reduction in hFUS observed in samples extracted from the adult heads of *GMR/*+;*hFUS/*+;*hsrω IR/*+ flies depends on some protein degradation process, the proteasome is not involved.

**Figure 6:**
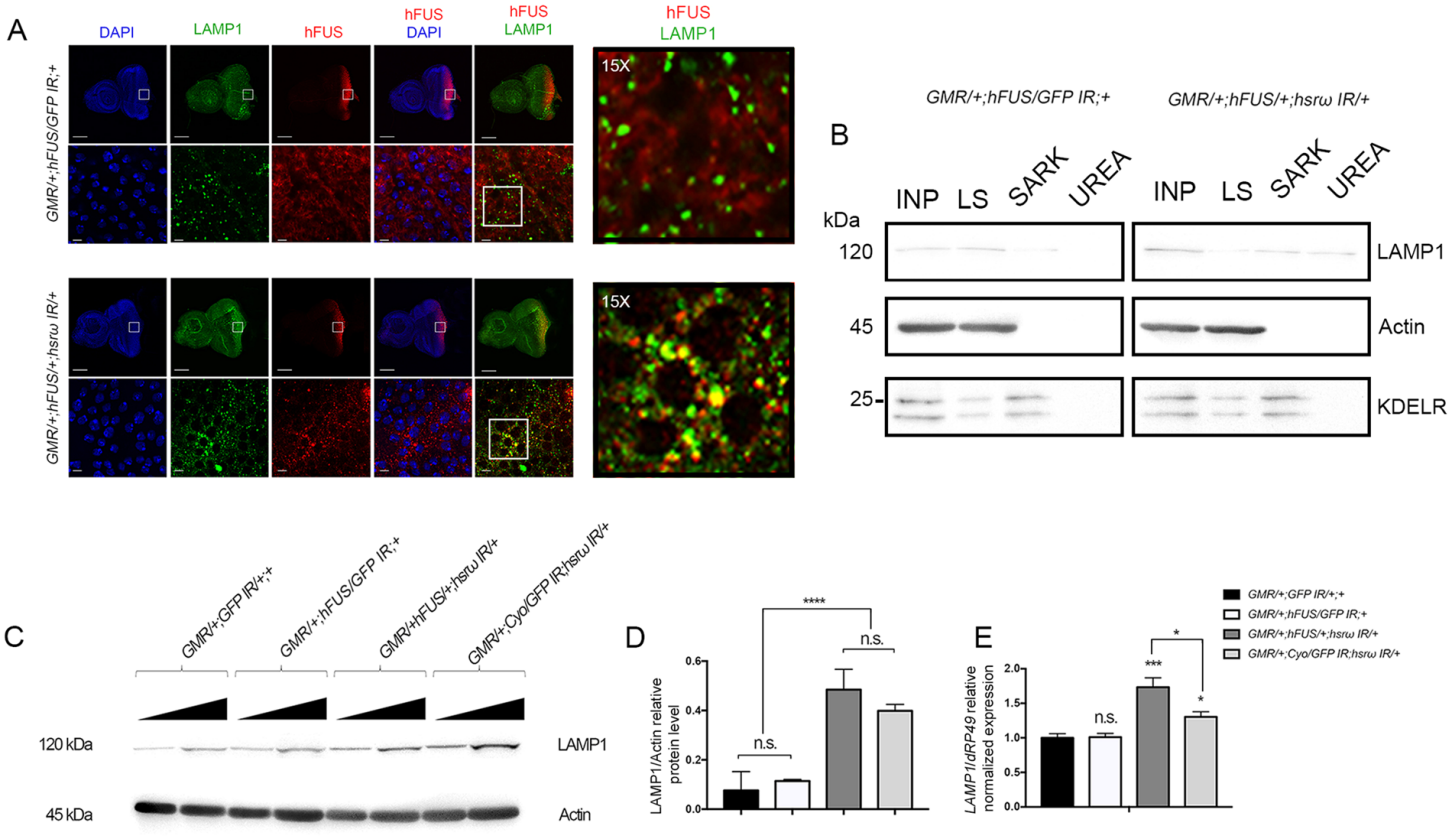
LAMP1 co-localizes with hFUS and is up-regulated by *hsrω* RNAi. (**A**) Double Immunofluorescence analyses for the hFUS (Red) and LAMP1 (Green) proteins were conducted using the eye imaginal discs of the third instar larvae of flies carrying *GMR/*+;*hFUS/GFP IR*;+ and *GMR/*+;*hFUS/*+;*hsrω IR/*+. Squares represent the area selected for a magnification in lower panels. The biggest white square marks the area for examination at higher magnification. DAPI staining DNA is shown in blue. False coloring and overlays were performed using Adobe Photoshop CS6 software. Fifteen samples were analyzed with a confocal laser-scanning microscope. The yellow spots are the resulting color of hFUS and LAMP1 co-localization. Larger and smaller scale bars indicate 150 and 10 μm, respectively. (**B**) A solubility test was performed with a sequential combination of low salt (LS), 2% N-Lauroylsarcosine-containing (SARK), and 8 M urea-containing (UREA) buffers. An Input (INP) was also loaded onto 10% SDS-PAGE in order to normalize the relative distribution of LAMP1 in the different fractions. The fractionation study of Actin and KDELR was carried out to understand whether all the proteins were properly fractionated and recovered. (**C**) A Western blot analysis was performed to examine LAMP1 abundance using an anti-LAMP1 antibody with proteins extracted with lysis buffer from the eye imaginal discs of the third instar larvae of different fly lines. Protein extraction was performed in duplicate from each fly line and two Western blots were independently performed for each sample. Ten and twenty micrograms of crude extracts were loaded onto 10% SDS-PAGE for each fly line. (**D**) Relative LAMP1 protein levels were measured relative to actin abundance. Gel plots were obtained with ImageJ32 and GraphPad Prism 7.0 software was used to statistically analyze data by calculating the significance of each measurement with respect to the control (*GMR/*+;*GFP IR/*+;+). ****p-value < 0.0001; n.s. = not significant. (**E**) *LAMP1* transcript levels were examined with RNA extracted from the third instar larvae of different fly lines at three independent times and this was followed by analyses with real-time RT-PCR in triplicate. The level of the *LAMP1* transcript was normalized with respect to *dRP49* abundance. Each quantified transcript is shown as the average of nine separate reactions for each fly line because real-time PCR was performed in triplicate with RNAs obtained from three independent extractions. GraphPad Prism 7.0 software was used to statistically analyze data by calculating the significance of each measurement with respect to the control (*GMR/*+;*GFP IR/*+;+). *p-value < 0.05; ***p-value < 0.001. n.s. = not significant.

We then examined the possible role of autophagy in hFUS degradation by examining the *in vivo* interaction of the *Drosophila* lysosomal marker LAMP1 with hFUS (Fig. 6). LAMP1 is the most abundant protein component of lysosomal membranes, a physiologically essential protein for the correct functions of lysosomes, and a widely used marker of autophagy^45^.

A double immunofluorescence analysis with the eye imaginal discs of the third instar larvae of flies carrying *GMR/*+;*hFUS/GFP IR*;+ and *GMR/*+;*hFUS/*+;*hsrω IR/*+ revealed the strong co-localization of these two proteins in flies co-expressing hFUS mRNA and *hsrω* dsRNA, while no interaction was found in the posterior region of the eye imaginal discs of flies expressing only the hFUS transgene (Fig. 6A). Moreover, by extending the study to the solubility of LAMP1 we found that in adult brain of flies carrying *GMR/*+;*hFUS/*+;*hsrω IR/*+ LAMP1 was also present in UREA fractions while no insoluble LAMP1 was detected in control (Fig. 6B), supporting the model of hFUS-LAMP1 inclusion formation upon depletion of *hsrω*.

Western blot analysis revealed a 2.86-fold increase in the expression of LAMP1 in both samples extracted with lysis buffer from adult heads of flies carrying *GMR/*+;*hFUS/*+;*hsrω IR/*+ and *GMR/*+;*Cyo/GFP IR/*+;*hsrω IR/*+, suggesting that the depletion of lncRNA *hsrω* was responsible for the augmentation of LAMP1 (Fig. 6C and D). The gene expression analysis with RNA extracted from the eye imaginal discs of third instar larvae suggested that the lncRNA *hsrω* is able to controls the abundance of *LAMP1* transcript (Fig. 6E).

Collectively, these results revealed that the down-regulation of the lncRNA *hsrω* through its RNAi not only reduced the abundance of the *hFUS* transcript, but also triggered the formation of hFUS-LAMP1 inclusion bodies, which may be degraded *via* autophagy.

### The formation of hFUS deposits may contribute to the titration of its toxicity

LAMP1 is transported to lysosomes, in which it is then degraded; thus, its augmentation indicates an increase in trafficking to lysosomes. The interaction of LAMP1 with hFUS in combination with a reduction in the amount of hFUS suggests a model in which *hsrω* RNAi induces the formation of hFUS inclusions and promotes its degradation *via* autophagosomes or autolysosomes.

The gene expression analysis of autophagy-related factors performed with RNA extracted from the eye imaginal discs of third instar larvae (Figure S7) revealed that autophagy may be induced in flies carrying *GMR/*+;*hFUS/*+;*hsrω IR/*+. The down-regulation of *mTOR* (0.71-fold) and up-regulation of *Atg1* and *Atg13* (1.32- and 1.26-fold) were observed. However, this analysis also revealed that GMR-driven hFUS expression alone promoted autophagy because *mTOR*, *Atg1*, and *Atg8* were mis-regulated. In flies carrying *GMR/*+;*hFUS/GFP IR*;+ , mTOR levels were 0.56-fold lower, while *Atg1* and *Atg8* levels were 1.63- and 1.54-fold higher than those in the control (Figure S7). Since hFUS transgene expression induces mitochondrial damage, as reported previously^17^, we herein hypothesized that the induction of autophagy in *GMR/*+;*hFUS/GFP IR*;+ may be a physiological response to apoptosis caused by mitochondrial damage dependent on hFUS expression, for example, a mitophagy process. Thus, in order to more precisely clarify whether autophagy is involved in the degradation of hFUS-LAMP1 inclusions, we employed other approaches such as genetic and drug screening methods using eye morphology as an indicator under an autophagy-inhibited background.

Genetic screening was performed by crossing the Atg8 loss-of-function mutant^46^ with flies expressing hFUS alone and co-expressing hFUS mRNA and *hsrω* dsRNA (Figure S8). No significant variation in the eye phenotype was observed because flies carrying *GMR/Atg8a* ^*mt*^;*hFUS/*+;+ and *GMR/Atg8a* ^*mt*^;*hFUS/*+;*hsrω IR/*+, in which autophagy is inhibited, showed aberrant and normal morphologies, respectively (Figure S8A), as observed with the corresponding flies in which autophagy was not inhibited (Fig. 3G and H). Up-regulated Ref(2)P/p62, examined by a Western blot analysis of proteins extracted from the eye imaginal discs of third instar larvae, confirmed the inhibition of autophagy in all *Atg8a* loss-of-function mutant flies (Figure S8B and C).

**Figure 7:**
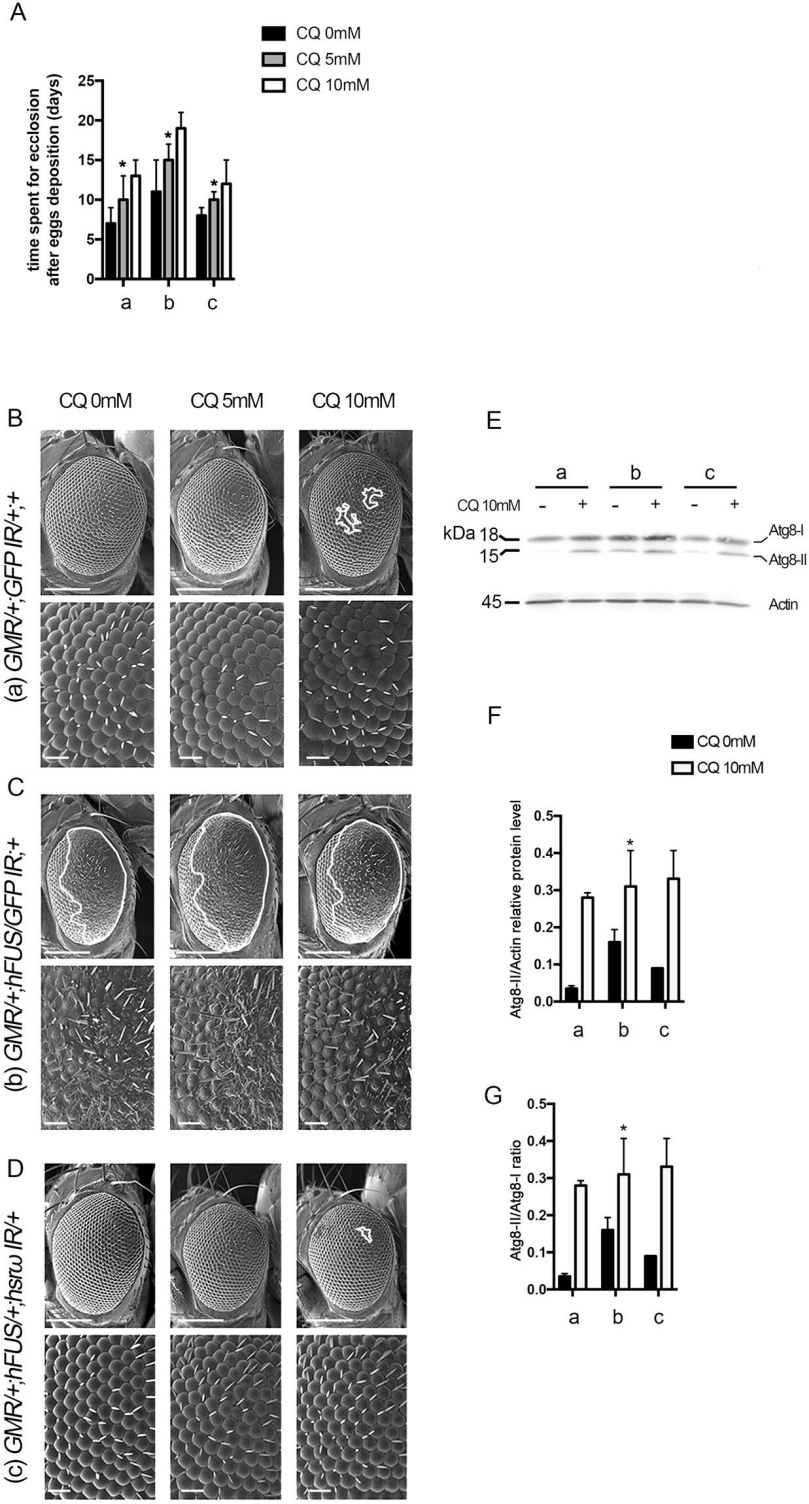
Inhibition of lysosomes does not affect the rescue of hFUS toxicity. **(A**) Chloroquine was used at 0 (CQ0), 5 (CQ5), and 10 (CQ10) mM to feed first instar larvae carrying a) *GMR-GAL4/*+;UAS*-GFP IR/*+;+ b) *GMR-GAL4/*+;UAS*-hFUS/*UAS*-GFP IR*;+ and c) *GMR-GAL4/*+;UAS*-hFUS/*+;UAS*-hsrω IR/*+. Twenty parental flies were crossed in each vial and triplicate crosses were performed for each genotype at each drug concentration. The time spent for eclosion after egg deposition was measured for each genotype and drug concentration. Eighty newborn flies were examined for each genotype. A statistical analysis was performed using GraphPad Prism 7.0 software. *p-value < 0.05. (**B–D**) A scanning electron microscope was used to examine the eye surface morphology of each fly line developed in the presence of the corresponding chloroquine concentration. White lines define the area of degeneration. The upper panels of each genotype show a 50-µm scale bar, while the middle panels represent a higher magnification of SEM (scale bar: 14.2 µm). (**E**) Ten micrograms of the crude extract was loaded onto 15% SDS-PAGE for each fly line. A Western blot analysis with an anti-Atg8a antibody was performed using protein extracted from the adult heads of a) *GMR-GAL4/*+;UAS*-GFP IR/*+;+ b) *GMR-GAL4/*+;UAS*-hFUS/*UAS*-GFP IR*;+ and c) *GMR-GAL4/*+;UAS*-hFUS/*+;UAS*-hsrω IR/*+ flies developed without (−) or with 10 mM chloroquine (+). Western blots were performed in duplicate, each with protein obtained by two independent extractions. Accordingly, Atg8aI (inactive) and Atg8II (active) showed molecular weights of 18 and 15 kDa, respectively. (**F**) The abundance of the active Atg8II protein was calculated relative to actin abundance. (**G**) The Atg8a active:inactive ratio was also measured. Gel plots of 15% SDS-PAGEs were obtained by ImageJ32 software and analyzed with GraphPad Prism 7.0 software.

Chloroquine is known to inhibit lysosome degradation through an increase in pH in lysosome vesicles. Two different concentrations of chloroquine (5 and 10 mM) were used to feed larvae and the compound eye morphology in the corresponding adult flies was then observed in comparison with untreated controls (Fig. 7). Since a delay in eclosion was observed proportionally to the increase in drug concentrations (Fig. 7A), we concluded that chloroquine was systemically absorbed by flies under our experimental conditions. However, even in this case, no significant variation in eye morphology was observed because flies carrying *GMR/*+;*hFUS/GFP IR*;+ and *GMR/*+;*hFUS/*+;*hsrω IR/*+ developed in the presence of chloroquine showed aberrant and normal eye morphologies, respectively, as noted with the corresponding untreated flies (Fig. 7B–D). The high level of active Atg8a (Atg8aII) measured by the Western blot analysis on protein extracted from the eye imaginal discs of third instar larvae further confirmed that chloroquine inhibited autophagy under our experimental conditions (Fig. 7E–G).

Collectively, these results revealed that autophagy was not involved in the degradation of hFUS-LAMP1 inclusions, even when it was induced in flies co-expressing hFUS mRNA and *hsrω* dsRNA. As described above, ubiquitin-negative hFUS-LAMP1 was not degraded by proteasomes, and we herein also demonstrated that they were not degraded by autophagy or lysosomes; therefore, none of the known protein degradation pathways appeared to be involved in the removal of hFUS-insoluble inclusions. In view of these results, we concluded that *hsrω* RNAi rescued hFUS toxicity by titrating its molar excess, which not only induced a reduction in the hFUS transcript, but also led to the formation of hFUS-LAMP1 inert inclusions. Hence, we propose a model in which LAMP1 is involved in targeting aggregation-prone proteins to form harmless deposits using a previously unknown mechanism that is *hsrω*-dependent and involves LAMP1.

### LAMP1 is necessary, but not sufficient to rescue hFUS toxicity

**Figure 8:**
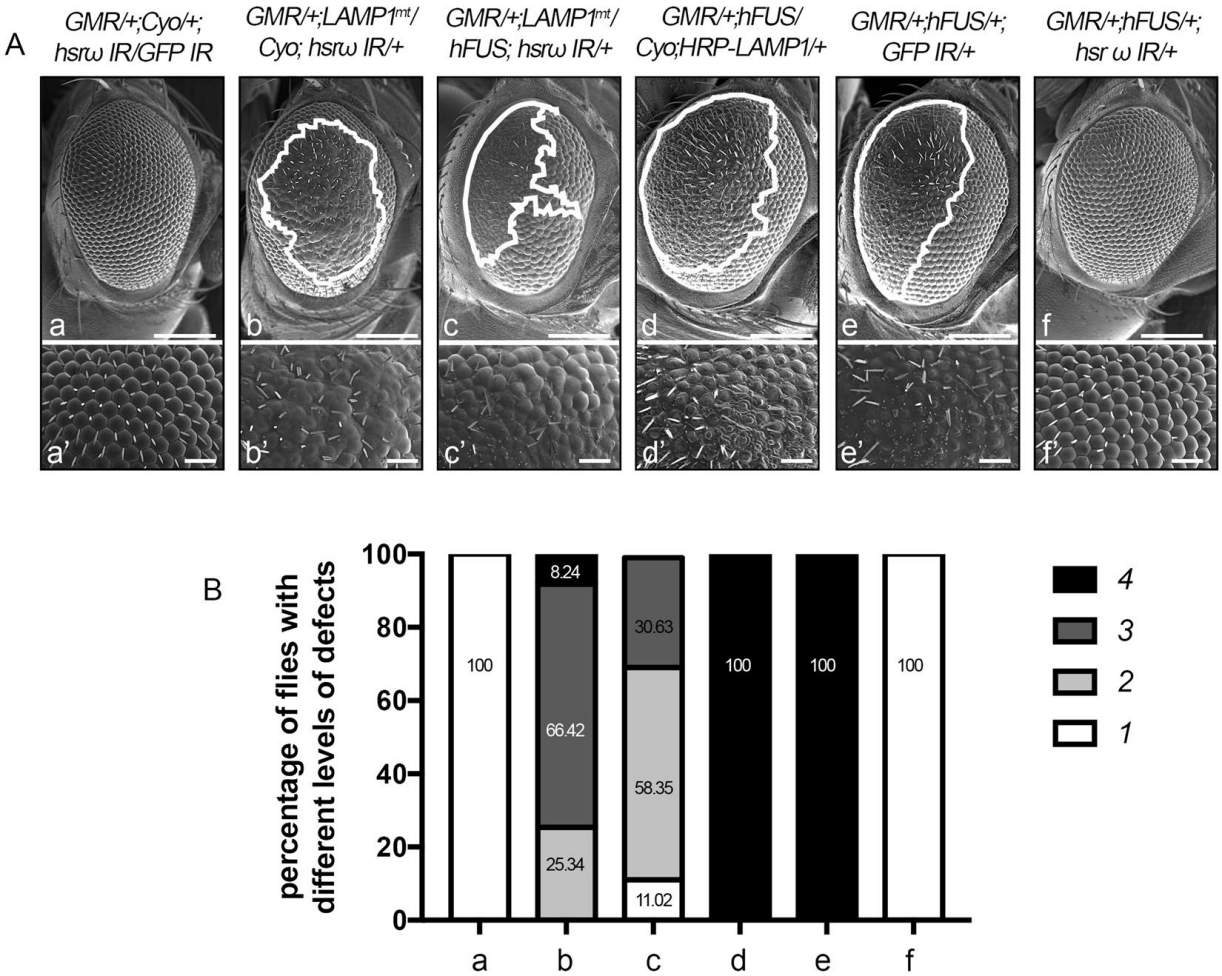
Role of LAMP1 in the rescue of hFUS toxicity. **(A**) The eye phenotype of 100 6-day-old flies for each line was examined under a light microscope, and the most representative ones were analyzed using SEM. White lines define the area of degeneration. Upper panels (a–f) show a 50-µm scale bar, while the middle panels (a’–f’) represent a higher magnification of SEM (Scale bar: 14.2 µm). **(B**) Four different phenotypes (1–4) were arbitrarily defined depending on the area of degeneration and pigmentation: (1) normal eyes; (2) small area of degeneration (less than 25% of the whole compound eye); (3) wide area of degeneration (more than 25% of the whole compound eye); (4) wide area of degeneration with the loss of pigmentation. The percentage of flies with different levels of degeneration were calculated for each genotype.

In order to definitively clarify whether LAMP1 plays a role in the control of hFUS toxicity, we performed a genetic crossing of the LAMP1 mutant with flies carrying *GMR/*+;*hFUS/*+;*hsrω IR/*+ and examined the adult compound eye morphology of progeny flies (Fig. 8). An experiment was also conducted to observe the external compound eye structure in adult flies co-expressing LAMP1 and hFUS (Fig. 8). A Western blot analysis was performed with an anti-LAMP1 antibody to compare protein levels in samples extracted from Canton S and LAMP1^MI15602^/Cyo adult heads. Quantification and statistical analyses revealed a 0.57-fold reduction in LAMP1 expression in flies heterozygous for the LAMP1^MI15602^ mutation (Figure S9A and B). The reduced expression of LAMP1 strongly enhanced the abnormal eye surface structure of flies expressing *hsrω* RNAi because 66.42% of flies carrying *GMR/*+;*LAMP1* ^*mt*^ */Cyo*; *hsrω IR/*+ showed a wider area of degeneration, while an apparently normal eye phenotype was observed in flies expressing *GMR/*+;*Cyo/*+;*hsrω IR/GFP IR* (Fig. 8A and B, compare a and b) and *LAMP1* ^*mt*^ */Cyo*;+ (data not shown). These results indicated that LAMP1 and the lncRNA *hsrω* genetically interacted. Moreover, the reduction in LAMP1 also affected the eye morphology of flies co-expressing hFUS mRNA and *hsrω* dsRNA because 58.35% and 30.63% of flies exhibited small and wide areas of eye degeneration, respectively, while flies carrying *GMR/*+;*hFUS/*+;*hsrω IR/*+ showed a rescued eye morphology (Fig. 8A and B, compare c and f).

These results confirmed that LAMP1 plays a role in the control of hFUS toxicity. However, GMR-driven LAMP1 overexpression in the hFUS background did not contribute to the amelioration of defects in the eye structures of flies carrying *GMR/*+;*hFUS/Cyo*;*HRP-LAMP1/*+ (Fig. 8A and B, compare d and e), suggesting that the rescue of hFUS toxicity driven by the depletion of *hsrω* does not only involve LAMP1. On the other hand, the lncRNA *hsrω* may be an upstream regulator of a network to control hFUS toxicity.

An analysis of LAMP1 expression was also performed (Figure S9). As described above (Fig. 6B–D), *hsrω* RNAi contributed to increasing the expression of LAMP1; therefore, a Western blot of protein extracted from the adult heads of flies carrying GMR*/*+;Cyo*/*+;*hsrω IR/*+ was used as a control to compare the expression of LAMP1 in proteins extracted from the adult heads of flies co-expressing *hsrω* dsRNA*-*LAMP1^mt^ and hFUS-LAMP1 (Figure S9). Accordingly, this analysis confirmed the genotypes of the flies examined, and, thus, LAMP1 was found to be down- and up- regulated in flies carrying *GMR/*+;*LAMP1* ^*mt*^ */Cyo*;*hsrω IR/*+ and *GMR/*+;*hFUS/Cyo*;*HRP-LAMP1/*+, respectively. Taken together, these results support LAMP1 being necessary, but not sufficient to rescue hFUS toxicity.

## Discussion

The main aim of the present study was to investigate the eventual role of lncRNAs in the pathomechanisms of FUS-proteinopathies. lncRNAs, which are a class of transcripts longer than 200 nucleotides with no or very low coding potential^47^ and similar to mRNA, but with proper specific features^48^, are predicted to function, often preferentially, in the nervous system, in which they may play roles in mediating neuronal development, behavior, and cognitive functions. The role of lncRNAs in neurological diseases is emerging as a new and important biological aspect and the number of publications in this field has rapidly increased over the past decade^49^. lncRNAs have been implicated in neurodegenerative diseases dependent on alterations in RNA metabolism because a large number of them are dysregulated upon the depletion of RNA-binding proteins causing FTLD and/or ALS, such as FUS and TDP43^27^. The formation of pathological inclusions of hnRNPs, which are a hallmark of these types of diseases, may aberrantly sequester important lncRNAs into pathological RNA-containing aggregates, thereby causing a global alteration in RNA processing^27^. From this perspective, the effects of lncRNAs in hnRNP-dependent neurodegenerative diseases are considered to be a secondary event caused by alterations in hnRNP abilities. Until now, it remained unclear whether lncRNAs induce alterations in the function of hnRNPs in order to drive toxicity.

We previously showed that the hnRNP dFUS is a new *hsrω*-interacting protein in *Drosophila* ^32^. Several other hnRNPs have the ability to bind to nuclear lncRNA *hsrω* to form ω-speckles, which are a specific class of nuclear bodies (NBs) involved in a wide range of cellular functions^50,51^. Notably, hFUS is a component of paraspeckles (one of the major classes of NBs), which are built by the lncRNA NEAT1^52^, and, as described above, it shows a specific ability to bind long transcripts.

Although global identity between hFUS and dFUS is less than 50%, the two proteins have highly similar specific domains^12^, and the transgenic expression of hFUS may completely rescue defects caused by the loss of dFUS^15^, thereby confirming its evolutionarily conserved function and indicating that hFUS in the fly also establishes a proper whole set of molecular interactions. However, we found that hFUS expressed in the fly showed a cytoplasmic distribution to exclude an interaction occurring with the lncRNA *hsrω* limited to the nucleus. Nevertheless, we revealed that *hsrω* RNAi induced a reduction in dFUS mRNA levels^32^ and negatively affected hFUS transcription. In the present study, we drove the expression of hFUS using a Gal4 system; therefore, it will be interested to examine whether the lncRNA *hsrω* has the ability to bind both *FUS* transcripts in order to stabilize the *FUS* mRNAs.

We herein found that the expression of hFUS in the fly induced a depletion of endogenous dFUS, an augmentation of the lncRNA *hsrω* and the alteration of dFUS40:dFUS34 ratio to increase the amount of insoluble dFUS34. Moreover, as we found through a fractionation studies, hFUS in the fly also shown the ability to form relative soluble aggregates. As such, when considering the FUS toxicity in fly models, all of these aspects should be taken into account. Cumulatively, the results herein reported contribute to clarify the relation of cause and effect underlying the hFUS toxicity. In fact, our studies support the idea that a depletion of endogenous dFUS upon expression of hFUS do not lead or contribute to hFUS toxicity because flies co-expressing hFUS and *hsrω* dsRNA show a normal eye phenotype despite the knockdown of *hsrω* is able to reduce the amount of dFUS^32^. In addition, a depletion of dFUS has been shown to cause a mild degeneration of adult eye compounds^41^.

To examine whether the augmentation of *hsrω* observed upon hFUS expression may contribute to hFUS toxicity, we studied the eventually effects of *hsrω* overexpression in a hFUS overexpression background. Interestingly, flies carrying *GMR/*+;*hFUS/*+;*EP93D/*+ shown normal adult eye compounds with a partial loss of pigmentation (Figure S10A). This result again strongly confirms the tight link between FUS and *hsrω* and supports the idea that the increased expression of *hsrω* upon hFUS expression in the fly may not contribute to hFUS toxicity. At the same time, these results suggest that the mechanism how *hsrω* knockdown can rescue the hFUS tocixity may be different from that triggered by *hsrω* overexpression. Further experiments are required to extend the knowledge in this regard. Notably, we herein did not examine whether *hsrω* indirectly affects *FUS* through the activity of other hnRNPs, which is possible because defects in the lncRNA *hsrω* alter compartmentalization^53,54^ and the functions^55^ of diverse hnRNPs that, in turn, have roles in RNA processing^56^.

Altogether our findings suggest that the hFUS amount may play a critical role in hFUS toxicity. For example, we found that hFUS toxicity was strongest at 28 °C. Notably, the transcriptional stimulation activity of GAL4 is known to be temperature dependent to drive the highest *UAS* expression. Moreover, we observed a severest eye degeneration in flies carrying *GMR*;+ ;*hFUS* than in flies carrying *GMR/*+;*hFUS/*+;*GFP IR/*+. On the lights of these results, we keep in view the hFUS toxicity in the fly could be a combination of events such as the formation of hFUS aggregates and the augmentation of insoluble dFUS34 which are both a result of hFUS expression. However, we herein did not study the role of insoluble dFUS34 whereby, further studies are needed in order to more fully understand the eventually impact of dFUS34 in hFUS-induced toxicity.

According to our results, we considered the first effective mechanism triggered by *hsrω* depletion to rescue hFUS toxicity to be a reduction in the amount of hFUS.

Notably, since the abundance of *hFUS* transcript was found high in flies co-expressing hFUS and *hsrω* to show an almost complete rescue (Figure S10B), further studies are required to clarify the mechanism how the overexpression of *hsrω* can be able to induce such a similar effect. In fact, the eye phenotype observed upon co-expression of hFUS and *hsrω* suggest that *hsrω* is able to modulate a protective mechanism to control the hFUS toxicity even in the presence of an exacerbated amount of hFUS.

When we performed a solubility test in the *hsrω* RNAi background, we found a marked shift in hFUS from soluble to insoluble because we fractionated hFUS only in 8 M Urea containing-buffer. A large number of studies agree that the toxicity of hFUS does not depend on its insolubility, however in ALS and FTLD human patients, the wt FUS is immunoreactive to ubiquitin in inclusion bodies, making it difficult to firmly establish whether the solubility shift is a prerequisite for pathogenesis. Since the formation of insoluble hFUS inclusions was associated with the rescue of an aberrant eye phenotype in the present study, we concluded that cytoplasmic hFUS inclusions are not toxic themselves and also raised a question regarding differences between pathological and non-pathological inclusions. Indeed, we herein demonstrated that insoluble hFUS in the *hsrω* RNAi background was negative for ubiquitin making the hFUS inclusions of this study not corresponding to the pathological inclusions observed in human FUS-proteinopathy patients. Moreover, the hFUS inclusions detected in this study were not attacked by proteasome and even when tagged by the lysosome-associated membrane protein LAMP1, hFUS inclusions were not degraded by autophagy or lysosomes. Recent studies reported that FUS forms distinct cytoplasmic partitions similar to inclusion protein deposits (IPODs), but with specific characteristics. These new cytoplasmic compartments are referred to as Interactor Specific Compartments/Inclusions (RISCI)^30^. However, RISCI have yet to be studied in *Drosophila*. Nevertheless, since FUS is an aggregation-prone protein, we speculate that a cellular mechanism to control the soluble-insoluble balance may exist in order to modulate the tendency of FUS to randomly form and sequester proteins in aberrant structures. On the other hand, *in vivo* studies showed that hFUS formed a specific class of non-toxic aggregates in a multi-step and RNA-dependent manner^37^. Cytoplasmic RNA granules are generally characterized by a set of markers i.e. TIAR, G3B1, and FMRP, in stress granules (SGs), but no RISCI’s marker has been characterized, so far. We assumed that LAMP1 is one of the specific factors targeting cytoplasmic FUS to form non-toxic partitions. Since hFUS cannot directly bind to *hsrω*, as previously explained, we considered the hFUS solubility shift to depend on a process caused by the depletion of *hsrω*. In addition, we revealed that LAMP1 was up-regulated by the expression of *hsrω* RNAi, and, thus, speculated that altered hFUS solubility is completely or partly attributed to the interaction with LAMP1. LAMP1 is needed in order to control hFUS toxicity because its depletion in flies co-expressing hFUS mRNA and *hsrω* dsRNA abolishes rescue, resulting in defects with diverse severities. However, due to the augmentation of LAMP1 only in hFUS-expressing flies, we were unable to rescue the aberrant phenotype. Two reasons have been proposed for this failure: LAMP1 may not have the ability to reduce the expression of hFUS, which supports the exacerbated amount of hFUS being the main factor inducing toxicity. Furthermore, other *hsrω*-dependent factors may be required for the formation of non-pathological hFUS deposits. Notably, the formation of hFUS-LAMP1 insoluble inclusions upon depletion of *hsrω* has been found in combination with the removal of hFUS relative soluble aggregates to support the model of hFUS aggregates play a significant role in hFUS toxicity. Therefore, we considered the second effective mechanism triggered by *hsrω* depletion to rescue hFUS toxicity to be the elimination of hFUS relative soluble aggregates trough the formation of LAMP1-targeted hFUS insoluble inclusions.

The novel results of the present study suggest a new *scenario*; a lncRNA-dependent mechanism may control the formation of non-toxic FUS inclusions. Under these conditions, the mis-regulation of some lncRNAs may not only be a consequence of FUS aggregate formation, as previously proposed, it may also be an instigating event of FUS-proteinopathies such as alterations in FUS expression and specific targeting/packaging into harmless deposits.

## Methods

### Fly stocks

Fly stocks were maintained at 25 °C on standard food containing 0.7% agar, 5% glucose, and 7% dry yeast. Canton S was used as the wt strain. Flies carrying *w*; *Sp/CyO*; UAS*-hsrω RNAi* (Bloomington, 59616) and + ;*UAS-93D* (Bloomington, 59614) were described previously^32,43^. Flies carrying + ;*UAS-GFP* were obtained from the Kyoto stock center (DGRC 107870). Flies carrying UAS*-GFP IR*;+ (41550), + ;UAS*-DIAP1* (6657), + ;UAS-*P35* (5073), *LAMP1* ^*MI15602*^ */Cyo*;+ (59738), and *Sp/Cyo*;UAS*-HRP-LAMP1* (42701) were obtained from the Bloomington stock center. The fly line *GMR-GAL4* was described previously^57^. The fly stock carrying the hFUS transgene was described previously^34^ and the Atg8a loss-of-function mutant fly was a gift from Juhász G.^46^.

### Light and scanning electron microscopy

One- and 6-day-old flies were anesthetized with 99% diethyl ether and the external surfaces of the eyes of corresponding flies were analyzed with the stereomicroscope, Olympus SZ61. Images were taken with Sony α NEX-5RY. In the examination using a scanning electron microscope, after anesthesia, adult flies were mounted on stages and inspected under the scanning microscope, SEM V-7800 (Keyence Inc.) in the low vacuum mode. The flies observed were developed at 25 or 28 °C. In the quantitative analysis of eye degeneration, we arbitrarily defined 4 categories: (1) normal eyes; (2) small area of degeneration (less than 25% of the whole compound eye); (3) wide area of degeneration (more than 25% of the whole compound eye); (4) wide area of degeneration with the loss of pigmentation. At least 100 adult flies of each phenotype were analyzed in each assay. ImageJ32 software was used to measure the area of the compound eye surface.

### Protein extraction and Western blotting

*Drosophila* adult flies were frozen in liquid nitrogen and the heads were separated by vigorous vortexing for 45 s. Crude extracts from the collected heads were obtained by homogenization in lysis buffer containing 10 mM Tris-HCl pH 7.5, 5 mM EDTA, 10% sucrose, 0.2% SDS, and protease inhibitors (Roche Diagnostic #11836170001) followed by centrifugation at 10000 x *g* at 4 °C for 20 min. Protein quantification was performed with Quick Start Bradford 1x Dye reagent (BIO-RAD). Proteins were separated by electrophoresis on SDS-polyacrylamide gels containing 10 or 15% acrylamide (SDS-PAGE) and then transferred to polyvinylidene difluoride membranes (Merck, Millipore, MA, USA). The blotted membranes were blocked with Tris-buffered saline/0.05% Tween-20 containing 5% skim milk at 25 °C for 1 h, followed by an incubation with appropriate primary antibodies: mouse polyclonal anti-Actin (1:1000, DSHB JL20); mouse monoclonal anti-KDEL Receptor (1:1000, Abcam ab69659); mouse monoclonal anti-hFUS (1:200, Santa Cruz sc-47711); rabbit polyclonal anti-dFUS antibody (1:2000^12^); rabbit polyclonal anti-LAMP1 (1:1000, Abcam ab30687); rabbit polyclonal anti-Atg8a (1:500, Creative Diagnostic CABT-B1670); mouse monoclonal anti-Hsp70 (1:2000, Abcam ab2787); rabbit polyclonal anti-Ubiquitin (1:1000, Cell Signal #3933); rabbit polyclonal anti-Ref(2)P (1:300, Abcam ab178440). After washing, the membranes were incubated with appropriate HRP-conjugated secondary antibodies: anti-rabbit and anti-mouse IgG (ThermoScientific, IL, USA), at 1:6000 and 1:5000 dilutions, respectively, at 25 °C for 1 h. Antibody binding was detected using ECL Western blotting detection reagents (Thermo Scientific) and images were analyzed using AE-9300 Ez-Capture MG (Atto).

### Immunohistochemistry

Eye imaginal discs were dissected from third instar larvae in 1X Phosphate-buffered saline (PBS), fixed in ice-cold 4% paraformaldehyde at 25 °C for 30 min, washed in PBS containing 0.3% Triton X-100 (PBT) 3 times for 10 min each, and blocked with 2% Normal Goat Serum (NGS) at 25 °C for 30 min. Samples were incubated with the following primary antibodies: mouse monoclonal anti-hFUS (1:50, Santa Cruz sc-47711), rabbit polyclonal anti-LAMP1 (1:100, Abcam ab30687), rabbit mono-specific synthetic peptide anti-cleaved caspase-3 (CC3) (1:00, Cell Signal Asp175-5A1E), and rabbit polyclonal anti-Hsp70 (1:100, Enzo ADI-SPA-812), at 4 °C for 16 h with agitation, and, after washing with PBT, they were treated with the fluorescent-conjugated secondary antibodies Alexa-488 anti-rabbit IgG (1:400, Invitrogen) and Alexa 594 anti-mouse IgG (1:400, Invitrogen) at 25 °C for 2 h. Staining with fluorescein isothiocyanate (FITC)-conjugated goat anti-Phalloidin (Pha) (1:1000, MP Biochemicals) was used at 25 °C for 30 min. All primary and secondary antibodies were diluted in PBT-2% NGS. Samples were mounted in Vectashield (Vector Laboratories-Inc.) and observed under a confocal laser-scanning microscope (OLYMPUS FLUOVIEW FV10i). Images were analyzed with MetaMorph Imaging System 7.7 (Molecular Devices Inc.).

### Solubility test

Total proteins were fractioned according to their solubility by adapting the protocol described previously^40^. One hundred *Drosophila* adult flies were frozen in liquid nitrogen, and the heads were separated by 45 s of vigorous vortexing and homogenized in 350 µl Low Salt buffer containing: 10 mM Tris, 5 mM EDTA, and 10% sucrose, pH 7.5. Homogenates were centrifuged at 2000 x *g* for 10 s. Fat and cuticles were discarded, and 50 µl of the homogenate was set aside as input (INP). The remaining 300 µl was centrifuged at 25000 x *g* at 4 °C for 30 min. The supernatant from this step was collected as the soluble fraction (LS). The pellet was further extracted with 150 µl ionic detergent buffer containing: 10 mM Tris, 5 mM EDTA, 2% N-Lauroylsarcosine (Sarkosyl), and 10% sucrose, pH 7.5 and then centrifuged at 180000 x *g* at 4 °C for 20 min. The supernatant from this step was collected as the SARK fraction. The remaining detergent-insoluble pellet was solubilized in 150 µl urea buffer containing 30 mM Tris, 8 M urea, 2 M thiourea, and 4% 3-[(3-Cholamidopropyl)dimethylammonio]-1-propanesulfonate hydrate (CHAPS), pH 8.5 and this fraction was designated as the UREA fraction. Protein concentrations of INP and LS fractions were determined by using Protein assay CBB solution (Nacalai Tesque) with BSA as a standard. For samples containing 2% N-Lauroylsarcosine, the Pierce™ BCA Protein Assay Kit (Thermo Fischer Scientific) was used due to detergent interference with the Bradford protein assay. The concentration of UREA fractions was determined by using Pierce 660 nm Protein Assay Reagent (Thermo Fischer Scientific). A total of 20 µg of each fraction was loaded onto 10 or 15% SDS-PAGE. Protein levels in individual fractions were normalized to input and referred in figures as “Percentage of protein fraction”. All buffers contained protease inhibitors (Roche Diagnostic) and were purified with a 0.45-µm filter.

### RNA extraction and qRT-PCR

Three replicates of 50 eye imaginal discs of third instar larvae were collected for each genotype. Homogenization was performed with a 1-ml syringe with a 27-G needle. Total RNA was isolated using an RNeasy Lipid Tissue Mini-Kit (Qiagen), followed by a DNAse treatment (DNAse I, Roche), and 0.5 µg of total RNA was reverse transcribed to cDNA using a Prime Script RT reagent kit (TaKaRa) according to the manufacturer’s instructions. RT-PCR was performed in triplicate for each single extraction with SYBR Green Master Mix: SYBR *Premix Ex Taq* II (Tli RNase H Plus) (TaKaRa) using the CFX96 Touch™ Real-Time PCR Detection System (BioRad), by the specific primer pairs listed in Supplementary Table S1. Samples were run in triplicate with SYBR^®^ Premix Ex Taq^TM^ II (TaKaRa) using CFX96 touch^TM^ (Biorad), and data were analyzed with a standard curve-based method calculated with CFX Manager^TM^ software. The specificity of primers was tested with melt curves created by CFX Manager^TM^ software and the agarose gel electrophoresis of amplified fragments. *dRP49* was used as an internal control.

### Chloroquine screening

Chloroquine (Sigma-Aldrich) as a 100-mM stock solution was freshly prepared in H_2_O and used to dissolve 3 g of instant *Drosophila* media (Carolina Biological Supply Company) at 0, 5, and 10 mM in a final volume of 4 mL for each vial. Parental flies were crossed in triplicate in each chloroquine-based vial at 28 °C under controlled humidity and light protection and removed three days later. Chloroquine solution was filled every four days according to the selected final concentration. Regarding each genotype, at least 10 representative male and female flies from at least two independent vials were selected for the external eye structure analysis with a scanning electron microscope.

### Statistical analysis

The Kruskal-Wallis test, followed by Dunnett’s multiple comparison test were used to statistically analyze differences among three or more groups. The Mann-Whiney U-test was used to compare data between two independent groups. The significance of differences between the variables was shown based on the p-value obtained: not significant when p > 0.05; *indicates p < 0.05; **indicates p < 0.005; ***indicates p < 0.001; ****indicates p < 0.0001. Values are presented as a mean and error bars indicate the standard deviation (SD).

## Electronic supplementary material

Supplementary figures
