# Supplementary material for: FUS toxicity is rescued by the modulation of lncRNA hsrω expression in Drosophila melanogaster
Source: Sci Rep. 2017 Nov 15;7:15660. doi: 10.1038/s41598-017-15944-y (PMC5688078; doi:10.1038/s41598-017-15944-y)
Supplement: Supplementary file 1 — Supplementary figures [file 41598_2017_15944_MOESM1_ESM.pdf]

FUS toxicity is rescued by the modulation of lncRNA *hsrω* expression in *Drosophila melanogaster*

Luca Lo Piccolo[1⊗], Salinee Jantrapirom[1], Yoshitaka Nagai[2], Masamitsu Yamaguchi[1]

[1]Department of Applied Biology, The Center for Advanced Insect Research, Kyoto Institute of Technology, Matsugasaki, Sakyo-ku, Kyoto 606-8585, Japan

[2]Department of Neurotherapeutics, Osaka University Graduate School of Medicine, 2-2 Yamadaoka, Suita, Osaka 565-0871, Japan

⊗corresponding author information: lucalopiccolo@gmail.com

Supplementary Figure S1

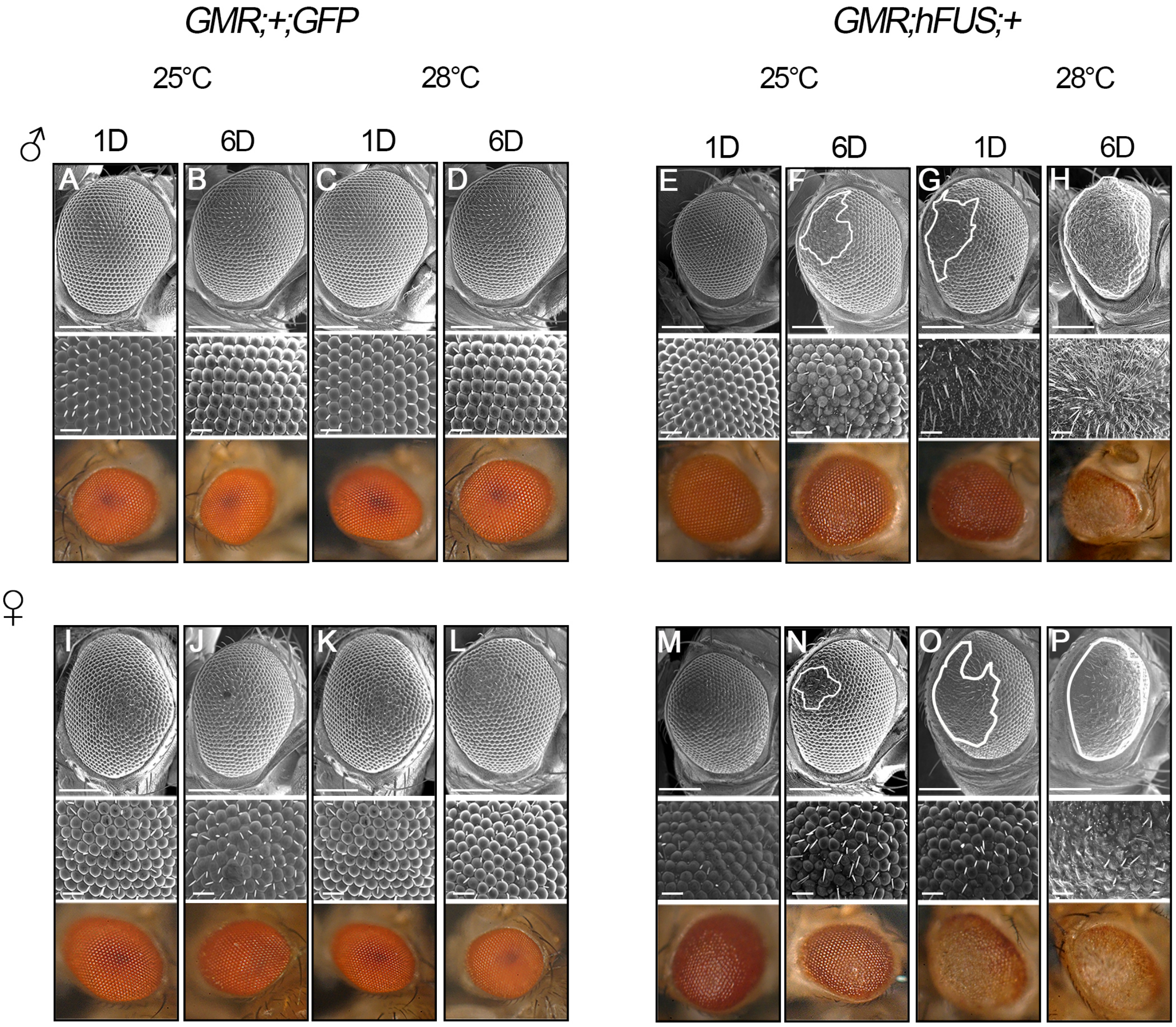

Supplementary Figure S1 Scanning electron microscope of hFUS expressing flies.
Scanning electron micrographs and light microscopy of compound eyes of 1 and 6 days-old flies carrying *GMR-GAL4;+:*UAS-*GFP* and *GMR-GAL4;*UAS *-hFUS;+* developed at 25 and 28 °C, respectively. The eye phenotype of 100 males and 100 females for each fly line was examined under light microscope and the most representative ones were analysed by using SEM. Scale bars in the upper panels of both males and females are 50µm; higher magnification is shown in the middle panels

(scale bar 14.2μm). Lowest panels show the eye observed with light microscope. Anterior is to the left and dorsal to the top. White lines define the area of degeneration.

Supplementary Figure S2

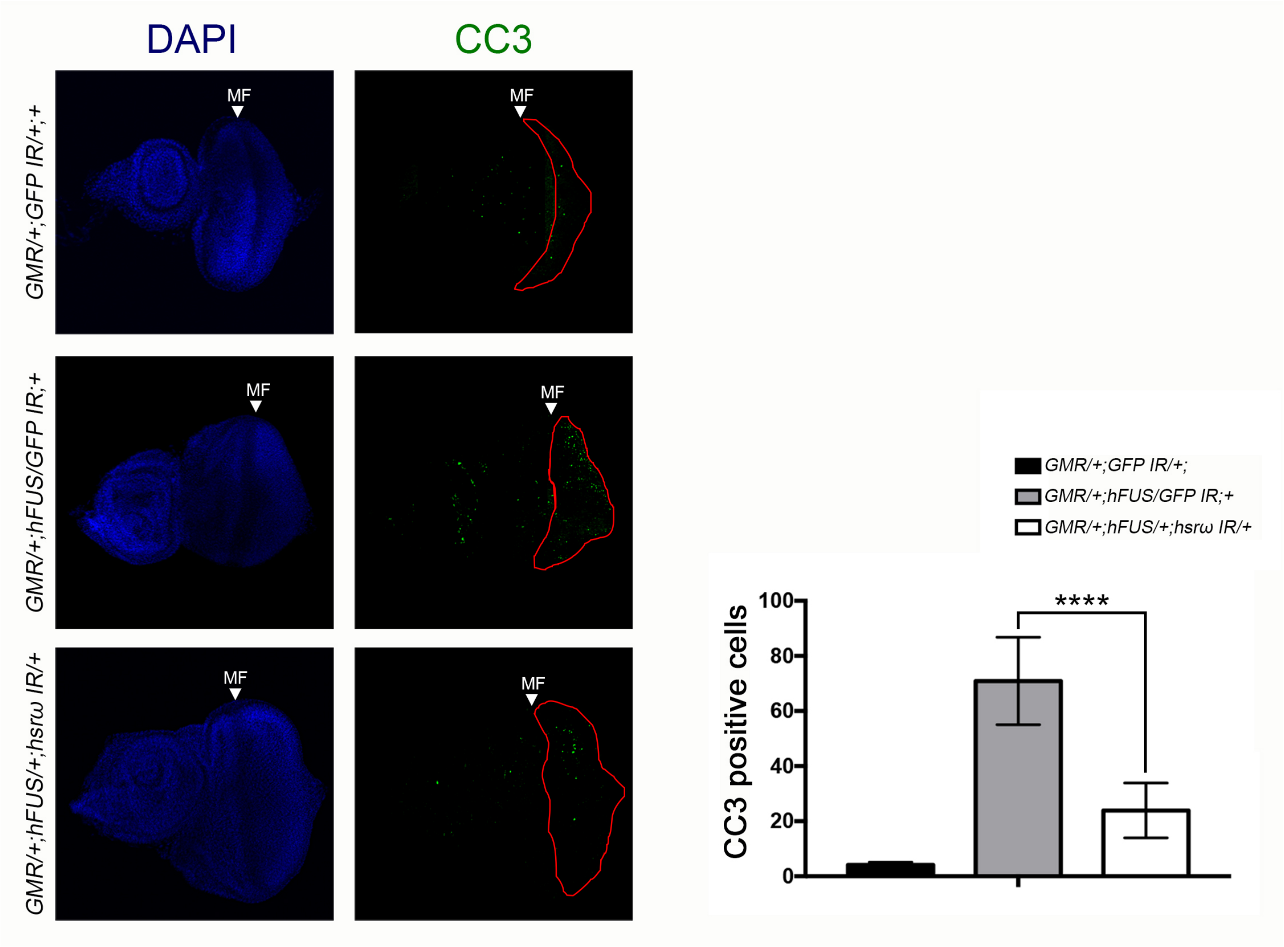

Supplementary Figure S2 *In vivo* staining of cleaved caspase 3.
Caspase activity was assayed by *in vivo* staining of cleaved caspase-3 (CC3) in eye imaginal discs of flies carrying *GMR-GAL4/+;*UAS-*hFUS/*UAS-*GFP IR;+* and *GMR-GAL4/+;*UAS-*hFUS/+;*UAS-*hsrω IR/+*. As control, flies carrying *GMR-GAL4/+;*UAS-*GFP IR/+;+* were used. A total of 10 imaginal discs of each genotype was observed by confocal laser-scanning microscope and CC3 positive cells were quantified in the posterior region. MF: morfogenetic furrow. The red lines define the area analysed by MetaMorph Image analysis software. Statistical analysis was performed by GraphPad Prism 7.0 software. n=10; ****=p-value<0.0001.

Supplementary Figure S3

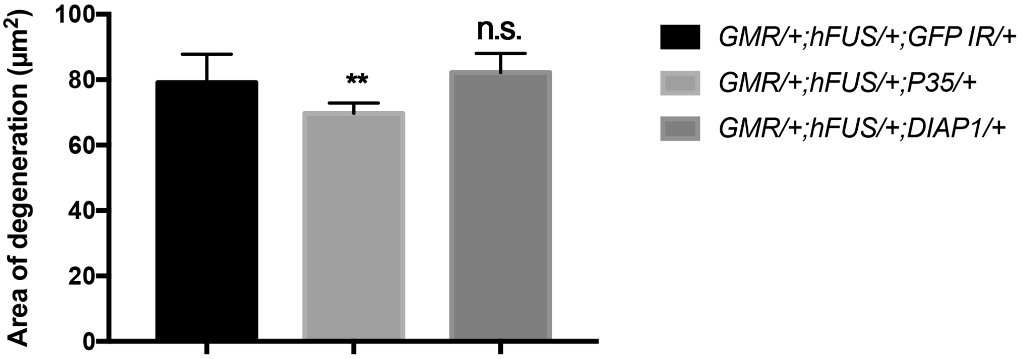

Supplementary Figure S3 Comparison of degenerated area in the eye surface of adult flies. The eye surface of 50 adult flies expressing hFUS (*GMR-GAL4/+;*UAS-*hFUS/+;*UAS-*GFP IR/+*) were examined by scanning electron microscope and the area of degeneration was measured (µm^2) by using ImageJ32 software. The eventual genetic interaction with P35 and DIAP1 apoptosis inhibitors was assayed by comparing the degenerated area of *GMR-GAL4/+;*UAS-*hFUS/+;*UAS-*GFP IR/+* with *GMR-GAL4/+;*UAS-*hFUS/+;*UAS-*P35/+* and *GMR-GAL4/+;*UAS-*hFUS/+;*UAS-*DIAP1/+*, respectively. Statistical analysis was carried out by GraphPad Prism 7.0 software. A total of 50 eyes for each genotype was examined. **=p-value<0.005; n.s.=not significant.

Supplementary Figure S4

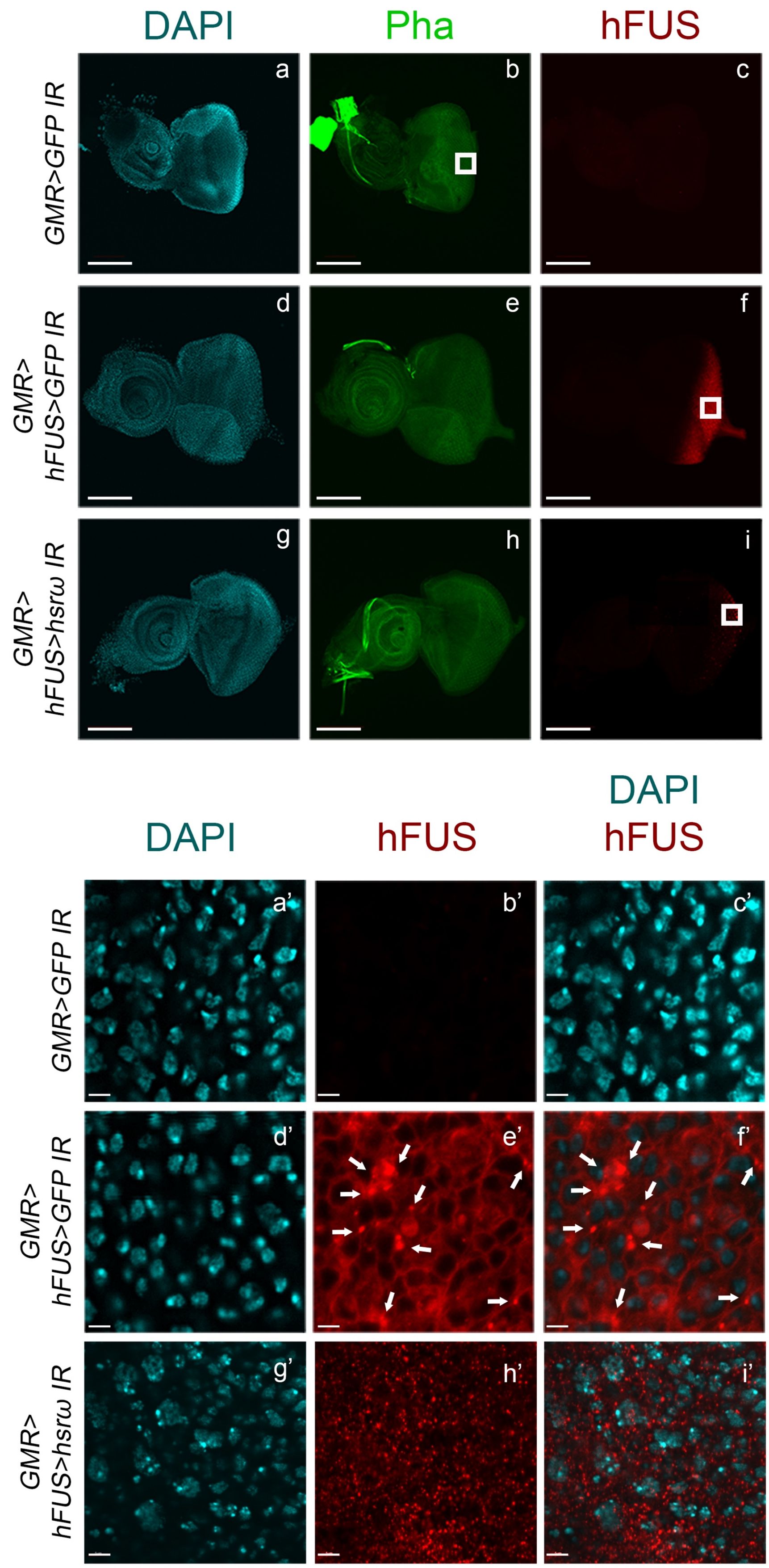

Supplementary Figure S4 hFUS sub-cellular localization.

In vivo staining of hFUS was performed in imaginal discs of third instar larvae of flies expressing hFUS in GMR>hFUS>GFP IR (*GMR-GAL4/+;*UAS-*hFUS/*UAS-*GFP IR;+*) and GMR>hFUS>hsrω IR (*GMR-GAL4/+;*UAS-*hFUS/+;*UAS-*hsrω IR/+*). GMR-driven expression of GFP IR was used as negative control (*GMR-GAL4/+;*UAS-*GFP IR/+;+*). A total of 10 eye imaginal discs for each genotype was examined with confocal laser-scanning microscope. No significant variation in hFUS sub-cellular localization was revealed. In the upper panels, white squares in the posterior region of imaginal disc were used to highlight the area of magnification further reported in the lower panels. White squares indicate the area examined with further higher magnification. White arrows show large spots of hFUS immuoreaction which may represent hFUS aggregates. Larger and smaller scale bar are 100 and 10μm, respectively.

Supplementary Figure S5

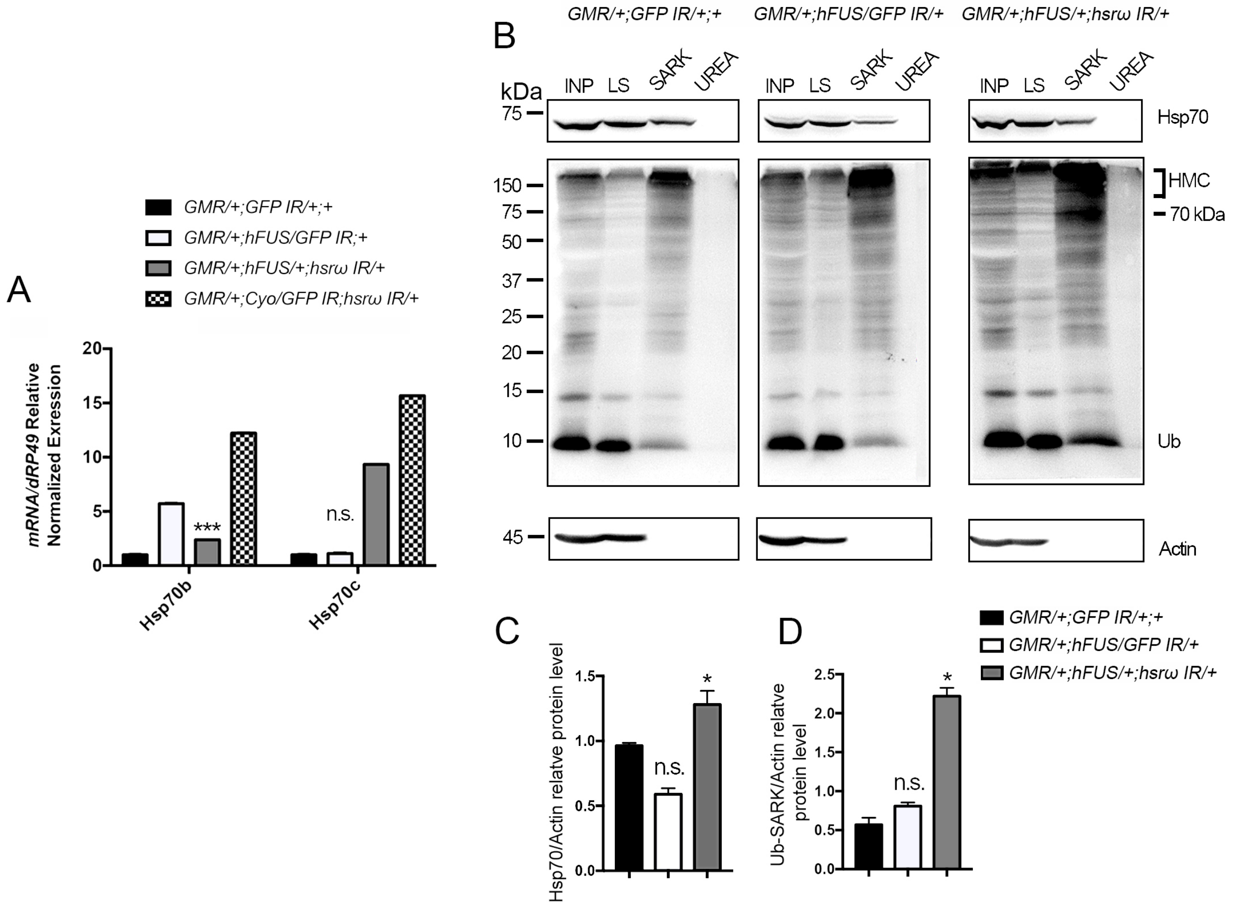

Supplementary Figure S5 Chaperons and Ubiquitin expression analysis.

A) Gene expression analysis of *Hsp70b* and *Hsp70c* was performed with real time RT-PCR by using RNA extracted from third instar larvae of flies carrying *GMR-GAL4/+;*UAS-*hFUS/*UAS-*GFP IR;+* and *GMR-GAL4/+;*UAS-*hFUS/+;*UAS-*hsrω IR/+*. The transcript abundance of these genes was also examined in RNA samples extracted from third instar larvae of flies carrying *GMR-GAL4/+;Cyo/*UAS-*GFP IR;*UAS-*hsrω IR/+*. The mRNA levels were normalized with respect to *dRP49* transcript. Each quantified transcript is the average of nine separate reactions for each fly lines, since a triplicate real time RT-PCR was performed with RNAs obtained from three independent extractions. GraphPad Prism 7.0 software was used to statistically analyse the data by calculating the significance of each measurement with respect to control (*GMR-GAL4/+;*UAS-*GFP IR/+;+*). *= p-value <0.05; ***= p-vale <0.001. n.s.= not significant. **B**) Western blot analyses were performed with anti-Hsp70 and anti-Ubiquitin antibodies, respectively by using proteins extracted with Low Salt (LS), 2% N-Lauroylsarcosine-containing (SARK) and 8M Urea-containing (UREA) buffers, respectively from adult heads of 6 days-old flies carrying *GMR-GAL4/+;*UAS-*hFUS/*UAS-*GFP IR;+* and *GMR-GAL4/+;*UAS-*hFUS/+;*UAS-*hsrω IR/+*, respectively. An Input (INP) was also loaded in the 10% SDS-PAGE to normalize the relative distribution of both proteins in the different fractions. **C-D**) The abundance of Hsp70 and Ubiquitin detected in INP and SARK, respectively were normalized with respect to Actin abundance. GraphPad Prism 7.0 software was used to statistically analyse the data by calculating the significance of each measurement with respect to control (*GMR-GAL4/+;*UAS-*GFP IR/+;+*). *= p-value <0.05; n.s.= not significant.

Supplementary Figure S6

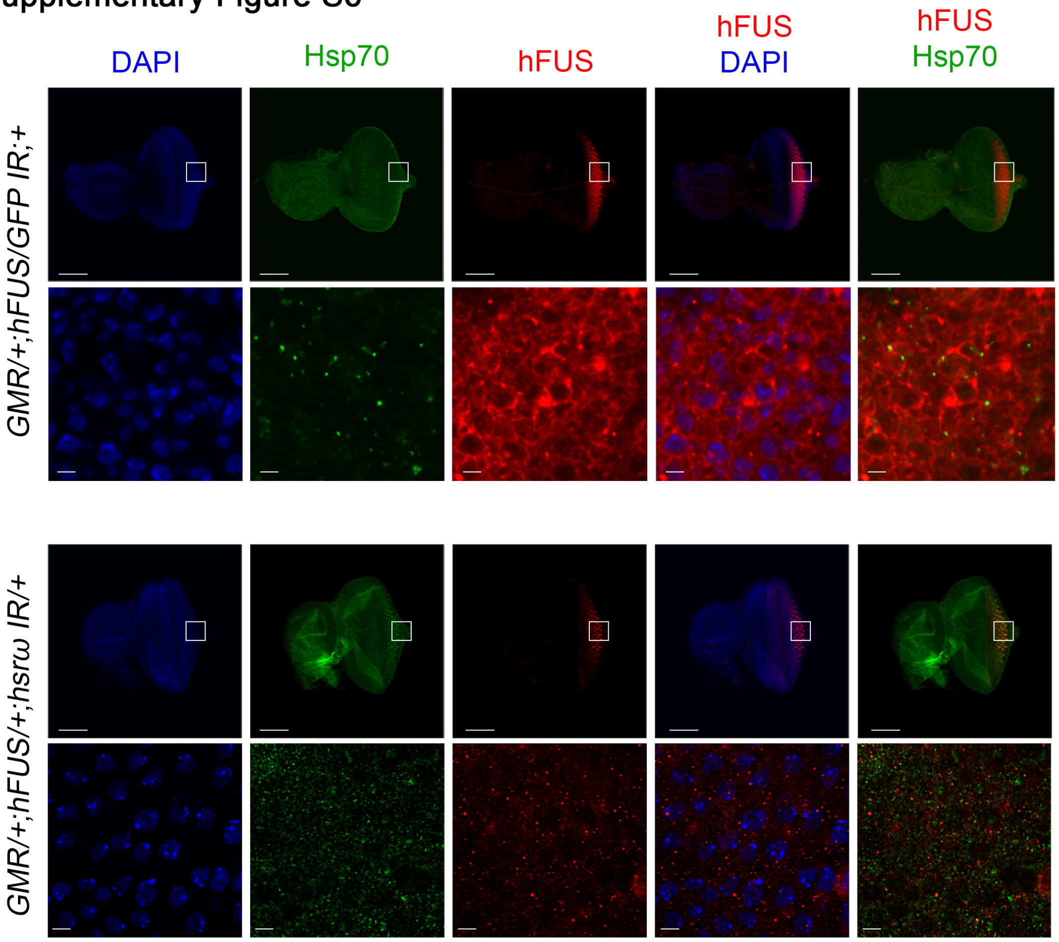

Supplementary Figure S6 *In vivo* double immunofluorescence of Hsp70 and hFUS.
Double Immunofluorescence for the hFUS (Red) and Hsp70 (Green) proteins was conducted in imaginal discs of third instar larvae of flies carrying *GMR-GAL4/+;*UAS-*hFUS/*UAS-*GFP IR;+* and *GMR-GAL4/+;*UAS-*hFUS/+;*UAS-*hsrω IR/+*, respectively. Squares represent area selected for higher magnifications showed further in each corresponding lower panel. DAPI staining DNA is shown in blue. False colouring and overlays were performed using Adobe Photoshop CS6 software. A total of 15 samples were analysed with confocal laser-scanning microscope. Larger and smaller scale bar indicate 150 and 10μm, respectively.

Supplementary Figure S7

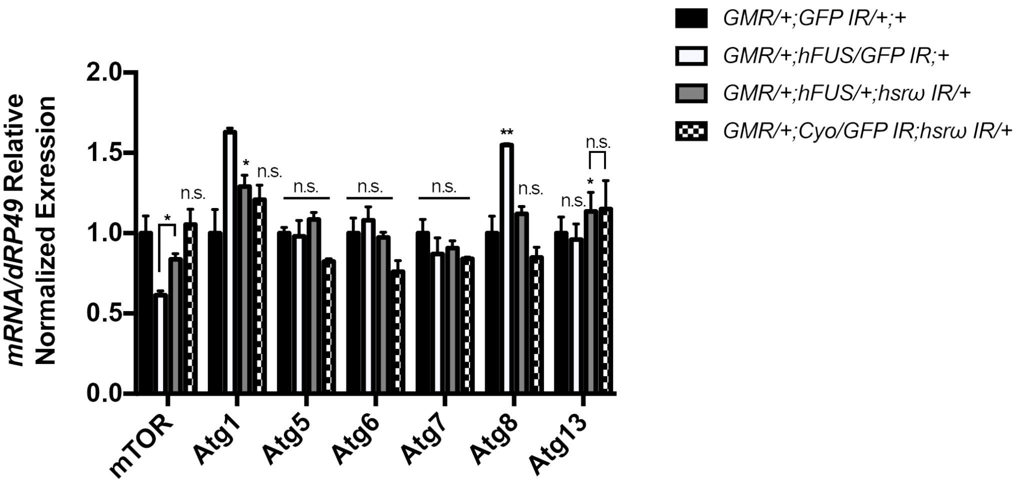

Supplementary Figure S7 Gene expression analysis of autophagy-related factors.
Gene expression analysis of *mTOR*, *Atg1*, *Atg5*, *Atg6, Atg7, Atg8* and *Atg13* was performed with real time RT-PCR by using RNA extracted from third instar larvae of flies carrying *GMR-GAL4/+;*UAS-*hFUS/*UAS-*GFP IR;+* and *GMR-GAL4/+;*UAS-*hFUS/+;*UAS-*hsrω IR/+*. The transcript abundance of these genes was also examined in RNA samples extracted from third instar larvae of flies carrying *GMR-GAL4/+;Cyo/*UAS-*GFP IR;*UAS-*hsrω IR/+*. The mRNA levels were normalized with respect to *dRP49* transcript. Each quantified transcript is the average of nine separate reactions for each fly lines, since a triplicate real time PCR was performed with RNAs obtained from three independent extractions. GraphPad Prism 7.0 software was used to statistically analyse the data by calculating the significance of each measurement with respect to control (*GMR-GAL4/+;*UAS-*GFP IR/+;+*). *= p-value <0.05; **= p-vale <0.005. n.s.= not significant.

Supplementary Figure S8

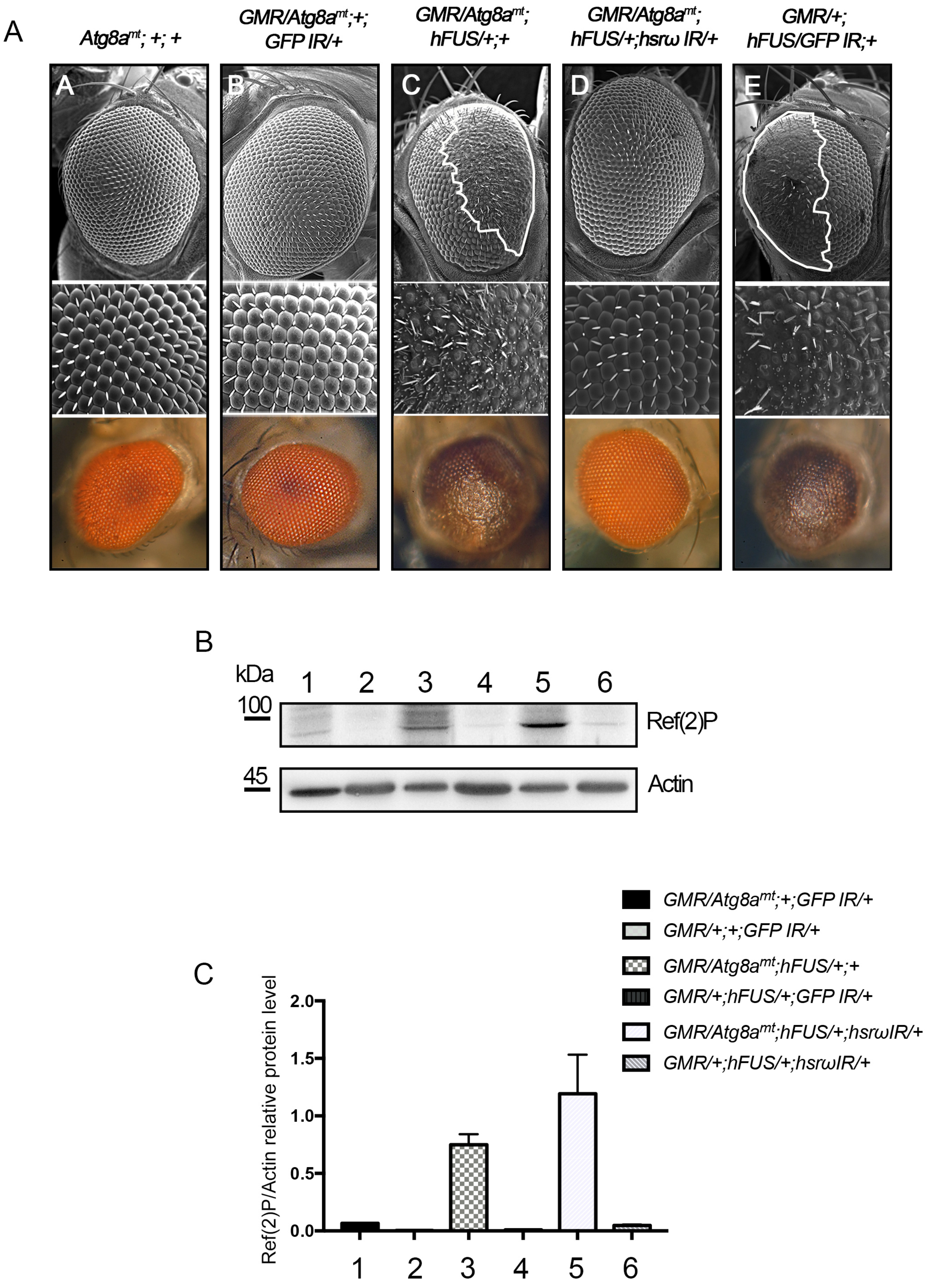

Supplementary Figure S8 Role of autophagy in the rescue of hFUS toxicity.

A) The eye phenotype of 80 adult flies expressing hFUS mRNA with and without *hsrω* double stranded RNA (dsRNA) in an inhibited-autophagy background were examined by scanning electron microscope and the area of degeneration was measured (µm^2) by using ImageJ32 software. Light microscope of the most representative eye of each fly line was used to show the pigmentation. Flies carrying *Atg8^{mt};+;+* and *GMR-GAL4/+;*UAS-*hFUS/*UAS-*GFP IR;+* were used as controls. White lines define the area of degeneration. **B-C**) Western blots analysis were performed with anti-Ref(2)P antibody by using protein extracted from adult heads of different fly lines. Two independent protein extraction were carried out. The abundance of Ref(2)P was normalized with respect to Actin abundance. GraphPad Prism 7.0 software was used to statistically analyse the data by calculating the significance of each measurement respect control (*GMR-GAL4/+;*UAS-*GFP IR/+;+*). Western blots were performed in duplicate for each protein extraction.

Supplementary Figure S9

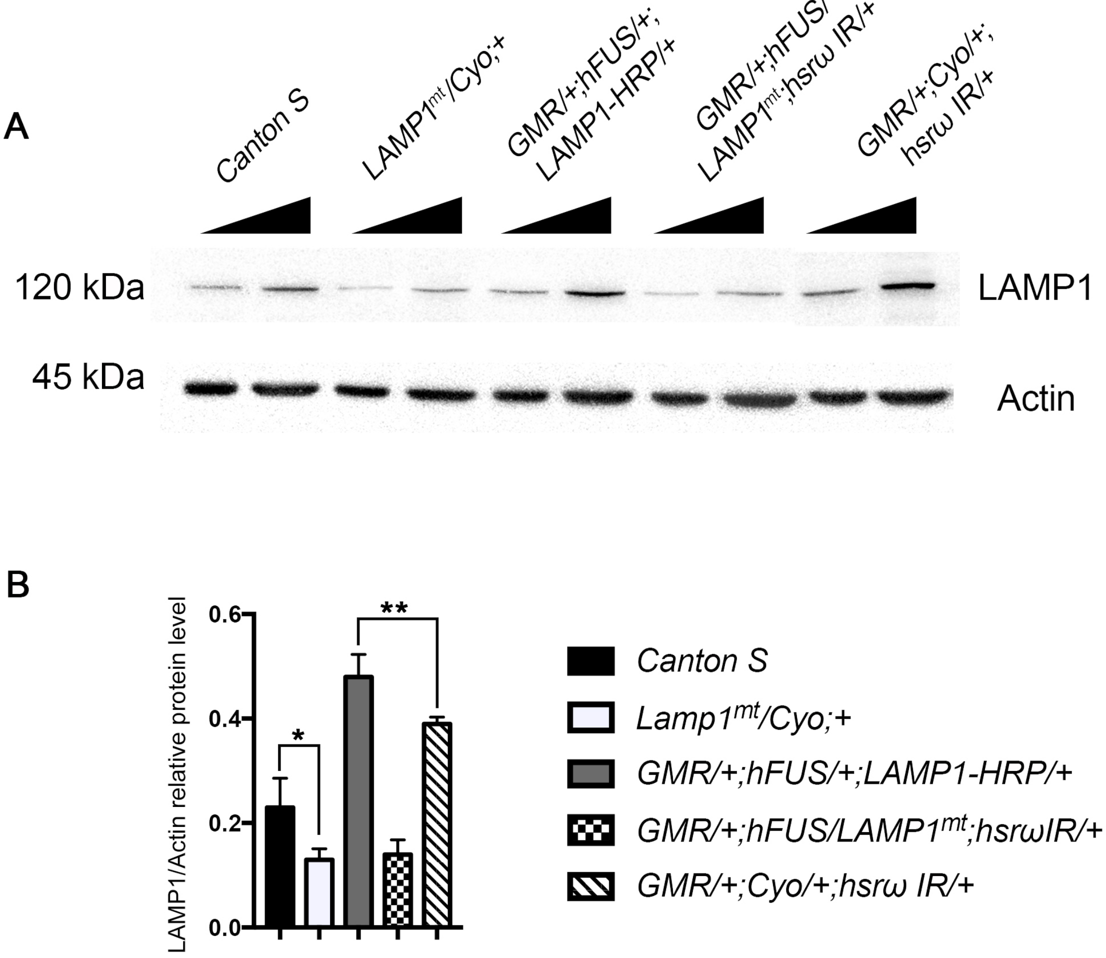

Supplementary Figure S9 Analysis of LAMP1 expression.
A) Western blots analysis were performed with anti-LAMP1 antibody by using protein extracted from adult heads of different fly lines. Two independent protein extractions were carried out. A total of 10 and 20 µg of crude extract were loaded in a 10% SDS-PAGE. **B**) The abundance of LAMP1 was normalized with respect to those of Actin. GraphPad Prism 7.0 software was used to statistically analyse the data by calculating the significance of LAMP1 measurement in flies carrying *LAMP1^{mt}/Cyo;+* with respect to Canton S control. Western blots were performed in duplicate for each protein extraction. *=p-value<0.05; **=p-value<0.005.

Supplementary Figure S10

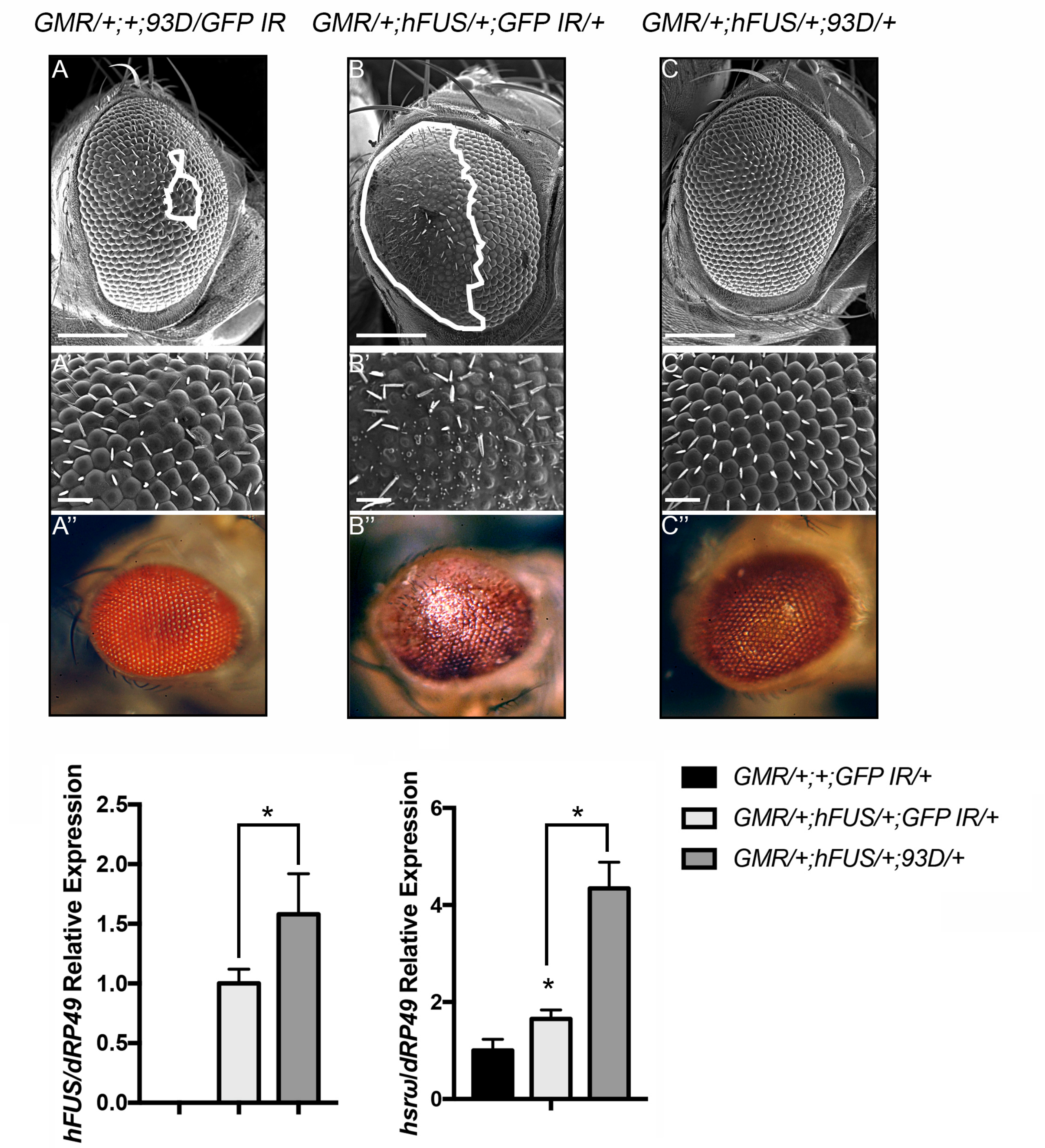

Supplementary Figure S10 Overexpression of the lncRNA *hsrω* rescues hFUS toxicity.
Scanning electron micrographs and light microscopy of adult compound eyes with the following genotypes: **A-A'')** *GMR-GAL4/+;+;*UAS-*93D/*UAS-*GFP IR,* **B-B'')** *GMR-GAL4/+;*UAS-*hFUS/+;*UAS-*GFP IR/+*, **C-C'')** *GMR-GAL4/+;*UAS-*hFUS/+;*UAS-*93D/+*. All flies were developed at 28°C. The eye phenotypes of approximately 100 female 6 days-old flies from each line were examined under a light microscope, and the most representative ones were analyzed using SEM. Upper panels **A-C** (scale bar 50µm). Middle panels **A'-C'** show a higher magnification (Scale bar:

14.2 µm). The lowest panels show the eye observed with a light microscope. No significant variation was observed in the eye phenotype among the individuals belonging to each line. Anterior is to the left and dorsal to the top. White lines define the area of degeneration. **D-E)** Quantification of transcript abundance. RNAs were extracted from the eye imaginal discs of the third instar larvae of different fly lines at three independent times and further analyzed by real-time RT-PCR in triplicate. The levels of the *hFUS* and *hsrω* transcripts were normalized with respect to *dRP49* abundance. Each transcript is the average of nine separate reactions for each fly line. A statistical analysis was performed using GraphPad Prism 7.0 software. *= p-value <0.05.

Supplementary Table S1 List of primers used in this work.

Gene name	Forward 5'-3'	Reverse 5'-3'
dRP49	AGATCGTGAAGAAGCGCACC	CGATCCGTAACCGATGTTGG
hFUS	CCTGGGCGAGAATGTTACAA	GGCTGTCCCGTCTTCTTATTT
hsrω	GAATTTCGCAATGCAGCAGG	GCCATTCGACACAGAGAGTAC
LAMP1	ATGGCGGCACAACTTAATTTTAC	TCTGGTCCCCAAATCAGATGAAT
Hsp70b	GGAGACACACACTTGGGCGGCGAG	TCTCGATGGTGGCCTCCGTGCTAG
Hsp70c	GAATCCCAACAACACGATCTTTGATG	AGGTAGGCCTCCGCGGTCTCTC
mTOR	TCCTGGAGGCACCAAACTTATC	GGAGCCACGGAGATTCTTCA
Atg1	CGTCTACAAAGGACGTCATCGCAAGAAAC	CGCCAAGTCGCCGCCATTGCAATACTC
Atg5	CCTGCGAATCTATACAGACGATGAC	AGCTCAGATGCTCGGACATCCATTG
Atg6	TGCACGCAATGGCGGAGTTATCTTTGC	CAGCTCCGCTTTCAGCTTAAAAGCAGC
Atg7	TGCCTTTCTGCTTCAGCAATGTCC	GGCCCCATTTTGCCATTTTTATTTAG
Atg8	TCGCAAATATCCAGACCGTGTGCCCGTC	GCCGATGTTGGTGGAATGACGTTGTTCAC
Atg13	CTGAAAACGACTGAGGGTGAC	GGCTGCCTTTAGGGTCTGG
